# Engineering the thin film characteristics for optimal performance of superconducting kinetic inductance amplifiers using a rigorous modelling technique

**DOI:** 10.12688/openreseurope.14860.2

**Published:** 2023-09-13

**Authors:** Boon-Kok Tan, Faouzi Boussaha, Christine Chaumont, Joseph Longden, Javier Navarro Montilla

**Affiliations:** 1 Department of Physics (Astrophysics), University of Oxford, Denys Wilkinson Building, Keble Road, Oxford, OX1 3RH, UK; 2 GEPI (CNRS UMR 8111), Observatoire de Paris, PSL Université, Paris, France

**Keywords:** Parametric Amplifier, Travelling Waves, Kinetic Inductance, Superconducting Thin Films, Bardeen-Cooper-Schrieffer (BCS) Theory, Transmission Lines, Microwave, Quantum-Limited Noise

## Abstract

**Background**: Kinetic Inductance Travelling Wave Parametric Amplifiers (KITWPAs) are a variant of superconducting amplifier that can potentially achieve high gain with quantum-limited noise performance over broad bandwidth, which is important for many ultra-sensitive experiments. In this paper, we present a novel modelling technique that can better capture the electromagnetic behaviour of a KITWPA without the translation symmetry assumption, allowing us to flexibly explore the use of more complex transmission line structures and better predict their performance.

**Methods**: In order to design a KITWPA with optimal performance, we investigate the use of different superconducting thin film materials, and compare their pros and cons in forming a high-gain low-loss medium feasible for amplification. We establish that if the film thickness can be controlled precisely, the material used has less impact on the performance of the device, as long as it is topologically defect-free and operating within its superconducting regime. With this insight, we propose the use of Titanium Nitride (TiN) film for our KITWPA as its critical temperature can be easily altered to suit our applications. We further investigate the topological effect of different commonly used superconducting transmission line structures with the TiN film, including the effect of various non-conducting materials required to form the amplifier.

**Results**: Both of these comprehensive studies led us to two configurations of the KITWPA: 1) A low-loss 100 nm thick TiN coplanar waveguide amplifier, and 2) A compact 50 nm TiN inverted microstrip amplifier. We utilise the novel modelling technique described in the first part of the paper to explore and investigate the optimal design and operational setup required to achieve high gain with the broadest bandwidth for both KITWPAs, including the effect of loss.

**Conclusions**: Finally, we conclude the paper with the actual layout and the predicted gain-bandwidth product of our KITWPAs.

## Plain language summary

A Kinetic Inductance Travelling Wave Parametric Amplifier (KITWPA) is a type of quantum amplifier developed recently that can potentially achieve high gain across broad bandwidth, with the lowest possible noise performance allowed by nature’s fundamental limits. In this article, we describe a new modelling technique that allows us to better capture the complex behaviour of the various materials used to form the amplifier. It also provides us with the flexibility to explore the use of more complicated structures in our amplifier to enhance their performance. As the amplifier is formed using the superconducting thin film, we investigate the behaviour of different types of superconducting material to compare their pros and cons in terms of magnitude of inductance that gives rise to high gain, and surface residual resistance that could potentially increase losses. We found that the choice of superconductor used is not critical to the performance of the amplifier, as long as the thickness of the film can be adjusted and fabricated within the capability of modern photo-lithography techniques. We therefore select one of the superconducting materials, the titanium nitride (TiN) film with appropriate thickness, for developing our KITWPAs. We further investigate the suitable transmission line topology required to form the amplifier. We then make use of the new methodology developed earlier to search for the optimum combination of various design and operational parameters required to operate the amplifier, paying special attention to the effect of loss that may impact the performance of our amplifiers. Finally, we conclude the article with the actual design of two KITWPAs, as well as their predicted gain profiles centred around 8 GHz with close to 100% fractional bandwidth performance.

## 1 Introduction

Many ultra-sensitive experiments such as astronomical receivers, quantum computation platforms and axion-like dark matter search experiments require the use of low-noise amplifiers to boost the signal-to-noise ratio of the weak detected signal. Traditionally, the amplification is achieved using High Electron Mobility Transistor (HEMT) amplifiers or low-noise Josephson Parametric Amplifiers (JPAs). Although the semiconductor-based HEMT amplifiers can achieve broadband amplification ^1– 3^, their noise performance is still far from approaching the quantum limit, and their heat dissipation is high, therefore limiting their deployment for large pixel count experiments due to the limited cryogenic cooling power capability. Whilst JPAs can achieve quantum noise performance, their operational bandwidth is relatively narrow at only a few percentage fractional bandwidth ^4– 6^. A travelling wave parametric amplifier (TWPA), on the other hand, can achieve similar high gain and low noise performance but with close to 2 *−* 3 *×* orders of magnitude wider bandwidth ^7, 8^. Their negligible heat dissipation and the ease of fabrication also makes them the preferred choice for large pixel count applications, such as enabling the construction of large millimetre-astronomical focal plane arrays ^9– 12^ and large qubit-number quantum computers ^13– 16^, and accelerating the searching speed of axion dark matter experiments ^17– 19^.

The operation of a TWPAs is similar to that of a JPA. The amplification is realised by transferring the energy from an injected strong modulating tone to the weak detected signal through a wave mixing mechanism within a nonlinear medium. In order to increase the interaction time between the strong pump and the weak signal to attain high gain, JPAs generally confine the nonlinear medium within a resonant cavity, hence the amplification only occurs within a limited bandwidth dictated by the cavity. TWPAs achieve broadband amplification by unfolding the nonlinear medium into a long transmission wire, thereby lifting the limitation imposed by the cavity. The source of the wire’s nonlinearity can be induced by either utilising the kinetic inductance of a superconducting wire (KITWPA) ^7, 16, 20– 28^ or with a series of Josephson junctions (JTWPAs) embedded along the wire ^8, 29– 35^.

Generally, the nonlinear reactance of the superconducting wire is weaker than that of the Josephson junction, hence KITWPAs require a much longer transmission length and higher pump power to achieve the same gain as the JTWPA counterpart. Even though the surface resistance of a short superconducting wire is negligible, a high current travelling along a much longer line spanning several tens to hundreds of wavelengths could still introduce a non-negligible compounded resistive loss. This resistive heating causes the KITWPA to have a poorer transmission strength and inferior noise performance compared to the JTWPA. Therefore it is important to carefully engineer the characteristics of the superconducting wire to achieve the quantum-limited noise performance with high gain over a shorter transmission length.

Although several designs of KITWPA have already been reported in the literature ^7, 16, 20– 27^, their choice of the thin film material used to form the superconducting wire and the topology of the transmission line are often not clearly clarified. They are most likely being dictated by the existing fabrication capabilities or from prior experience in the design of superconducting devices that require high kinetic inductance such as microwave kinetic inductance detectors (MKIDs). The lack of extensive investigation of the effect of using different materials and transmission line topologies therefore indicates that the performance of the KITWPA may not yet have been fully optimised. Therefore, in this paper, we aim to explore the effect of the properties of different types of superconducting thin films and transmission line topologies on the performance of the KITWPA, in particular their impact on the gain and loss performance of the amplifier, and the limits where the amplifier may cease to operate due to high resistive loss. Based on these analyses, we present the designs of two optimal KITWPAs that can achieve low loss performance with the shortest possible transmission length. We show the gain-bandwidth performance of the KITWPAs predicted using a novel method we developed to rigorously simulate the performance of the amplifiers taking advantage of 3-D electromagnetic ( *em*) simulators and the simplicity of coupled-mode equations taking into account losses and subtle *em* effects. We shall discuss this new method in detail in the subsequent sections.

## 2 Methods

A TWPA relies on the nonlinear behaviour of a transmission medium to mediate energy exchange between several different frequency components through a wave mixing process. Amplification can be achieved through varying the nonlinear reactance of the medium by applying a strong ‘pump’ current at frequency *ω*_p_. If a weak signal at frequency *ω*_s_ is applied, under proper conditions, the time-varying reactance would transfer the power from the pump to the signal, thereby achieving amplification. In theory, because this mechanism relies purely on nonlinear reactance response, the dissipation can be made arbitrarily small and Johnson noise can be eliminated, allowing the quantum limit to be reached. During the mixing process, a third frequency component, the ‘idler’ at *ω*_i_ will be generated and amplified to preserve the total momentum of the system. To a good approximation, these nonlinear inductances vary quadratically with current, therefore enabling the provision of wave-mixing through the nonlinear relation between the inductance and the RF currents.

The parametric gain produced through wave-mixing can be calculated using the coupled-mode differential equations ^36– 38^, and is governed primarily by the phase relation of the waves propagating down the line. The gain is maximised if the phases of all propagating waves including the idler are matched. Under this condition, the signal would grow exponentially with line length and pump power instead of quadratically. However, these superconducting nonlinear media are generally dispersive, therefore a major challenge in designing the TWPAs is the need for a dispersion control element that can phase-match all the waves propagating along the parametric gain medium. These phase-matching elements can be realised e.g., in the form of a series of shunt resonators coupled to the nonlinear transmission line termed the resonator phase-matching (RPM) scheme ^8, 31^, or by making use of periodical loading elements along the transmission medium ^7, 28, 34^ to create multiple abrupt stopbands along the frequency axis. The wave vector near these regions diverge exponentially away from the otherwise linear dispersion relation, similar to those found in the photonic crystals or electrons in a periodic potential. By placing the pump near one of these divergence regions, the phase of the pump can be shifted to minimise the phase mismatch, and allow the maximum energy transfer from the pump to the signal. They can also be engineered such that the additional stopbands coincide with the frequency of the higher pump harmonics generated through self-modulation, hence prohibiting their propagation and prevent shock wave formation.

The parametric gain produced through wave-mixing in KITWPA can be described using a set of coupled mode equations. The nonlinear wave equation that governs the operation of the amplifier is given by:


$$\frac{\partial^{2}I}{\partial z^{2}}=RGI+(LG+RC)\frac{\partial I}{\partial t}+C\frac{\partial }{\partial t}\left[L\frac{\partial I}{\partial t}\right]\;(1)$$

where *R*, *L*, *G* and *C* are the resistance, inductance, conductance and capacitance per unit length, respectively, *I* the propagating tones and *z* the position along the transmission line.

The nonlinear inductance term can be approximated by a quadratic expansion ^7, 39^ as *L*( *I*) = *L*_0_ [1 + ( *I/I*_0_) ^2^], where *L*_0_ is the intrinsic inductance and *I*_0_ is the scale of nonlinearity related to the critical current value *I*_C_ of the film. The solution for Equation 1 can be obtained by assuming the propagating fields are comprised of a group of forward moving waves represented by *I*( *z*, *t*) = $\frac{1}{2}A_{\text{m}}(z)e^{\left(-\gamma_{\text{m}}z+\omega_{\text{m}}t\right)}$ +c.c., where m=p,s,i corresponding to the pump, signal and idler wave respectively, *γ*_m_ = *α*_m_ + *iβ*_m_ the complex propagation constant comprising the attenuation constant *α* and the wave vector *β*, *A*_m_ the slowly varying amplitudes of the waves, *ω*_m_ the angular frequencies and c.c. represents the complex conjugate terms. Here we assume an efficient suppression of the pump’s harmonics via schemes that would be explained in later section, hence we ignore all the higher harmonic components in our calculation. For operation in the four wave mixing (4WM) regime, the relation between the pump, signal and idler is dictated by 2 *ω*_p_ = *ω*_s_ + *ω*_i_.

Substituting the propagating waves *I*( *z*, *t*) into Equation 1, assuming that $\left|\frac{d^{2}A_{\text{m}}}{dz^{2}}\right|\ll \left|\gamma_{\text{m}}\frac{dA_{\text{m}}}{dz}\right|,$ and $\left|\frac{dA_{\text{m}}}{dz}\right|\ll \left|\gamma_{\text{m}}A_{\text{m}}\right|$ 7, and collecting the terms that have the same frequency as the pump, signal and idler produces a set of coupled equations in the low-loss limit ( *R* « ω *L* and *G* « ω *C*):


$$\begin{array}{l} \gamma_{\text{p}}\frac{\partial A_{\text{p}}(z)}{\partial z}=\frac{\beta_{\text{p}}^{\text{2}}}{8I_{\text{0}}^{\text{2}}}[A_{\text{p}}A_{\text{p}}A_{\text{p}}^{\text{*}}e^{-2\alpha_{\text{p}}z}+2A_{\text{p}}A_{\text{s}}A_{\text{s}}^{\text{*}}e^{-2\alpha_{\text{s}}z}\\ \;+2A_{\text{p}}A_{\text{i}}A_{\text{i}}^{\text{*}}e^{-2\alpha_{\text{i}}z}+2A_{\text{p}}^{\text{*}}A_{\text{s}}A_{\text{i}}e^{-(\alpha_{\text{s}}+\alpha_{\text{i}})z}e^{{\text{Δ}}_{\beta }z}]\\ \gamma_{\text{s}}\frac{\partial A_{\text{s}}(z)}{\partial z}=\frac{\beta_{s}^{\text{2}}}{8I_{\text{0}}^{\text{2}}}[2A_{\text{p}}A_{\text{p}}^{\text{*}}A_{\text{s}}e^{-2\alpha_{\text{p}}z}+A_{\text{s}}A_{\text{s}}A_{\text{s}}^{\text{*}}e^{-2\alpha_{\text{s}}z}\\ \;+2A_{\text{s}}A_{\text{i}}A_{\text{i}}^{\text{*}}e^{-2\alpha_{\text{i}}z}+A_{\text{p}}A_{\text{p}}A_{\text{i}}^{\text{*}}e^{(-2\alpha_{\text{p}}+\alpha_{\text{s}}-\alpha_{\text{i}})z}e^{-{\text{Δ}}_{\beta }z}]\\ \gamma_{\text{i}}\frac{\partial A_{\text{i}}(z)}{\partial z}=\frac{\beta_{i}^{\text{2}}}{8I_{\text{0}}^{\text{2}}}[2A_{\text{p}}A_{\text{p}}^{\text{*}}A_{\text{i}}e^{-2\alpha_{\text{p}}z}+2A_{\text{s}}A_{\text{s}}^{\text{*}}A_{\text{i}}e^{-2\alpha_{\text{s}}z}\\ \;+A_{\text{i}}A_{\text{i}}A_{\text{i}}^{\text{*}}e^{-2\alpha_{\text{i}}z}+A_{\text{p}}A_{\text{p}}A_{\text{s}}^{\text{*}}e^{(-2\alpha_{\text{p}}-\alpha_{\text{s}}+\alpha_{\text{i}})z}e^{-{\text{Δ}}_{\beta }z}] \end{array}\;(3)$$

where ∆ *_β_* = 2 *β*_p_ − *β*_s_ − *β*_i_. This set of coupled differential equations can be solved numerically to probe the evolution of the wave amplitudes of the pump, signal and idler, hence the gain or attenuation of these waves along the transmission line. One can prove that under the undepleted pump assumption i.e., the pump amplitude remains unchanged throughout the transmission length *A*_p_( *z*) = *A*_p_(0) where *A*_p_(0) is the initial pump amplitude, the parametric gain $g=\frac{1}{2}\sqrt{\kappa_{\text{s}}\kappa_{\text{i}}-{\text{Δ}}_{\phi }}$ is maximised when the total phase mismatch ∆ _*ϕ*_ = 2 *ϕ*_p_ − *ϕ*_s_ − *ϕ*_i_ + ∆ _*β*_ = 0 where $\phi_{\text{p}}=\frac{\beta_{\text{p}}}{8I_{\text{0}}^{\text{2}}}A_{\text{p}}{\left(0\right)}^{2}$ , $\phi_{\text{s,i}}=\frac{2\beta_{\text{s,i}}}{\beta_{\text{p}}}\phi_{\text{p}}$ and $\kappa_{\text{s,i}}=\frac{\phi_{\text{s,i}}}{2}$ . Nevertheless, in all the analyses presented in the following, we do not include the assumption of undepleted pump to highlight the effect of the additional loss term α on the performance of the KITWPAs.

A KITWPA can also be designed to operate in the three wave mixing (3WM) regime by DC-biasing the nonlinear transmission line ^20, 23, 40, 41^. In this case, *ω*_p_ = *ω*_s_ + *ω*_i_, and the DC-biased 3WM coupled mode equation can be derived using the same procedure, given as:


$$\begin{array}{l} \gamma_{\text{p}}\frac{\partial A_{\text{p}}(z)}{\partial z}=\frac{\beta_{\text{p}}^{\text{2}}}{8I_{\text{0}}^{\text{2}}}[A_{\text{p}}A_{\text{p}}A_{\text{p}}^{\text{*}}e^{-2\alpha_{\text{p}}z}+2A_{\text{p}}A_{\text{s}}A_{\text{s}}^{\text{*}}e^{-2\alpha_{\text{s}}z}\\ \;+2A_{\text{p}}A_{\text{i}}A_{\text{i}}^{\text{*}}e^{-2\alpha_{\text{i}}z}+4I_{\text{dc}}^{2}A_{\text{p}}\\ \;+4I_{\text{dc}}A_{\text{s}}A_{\text{i}}e^{-(\alpha_{\text{s}}+\alpha_{\text{i}})z}e^{{\text{Δ}}_{\beta 3}z}]\\ \gamma_{\text{s}}\frac{\partial A_{\text{s}}(z)}{\partial z}=\frac{\beta_{s}^{\text{2}}}{8I_{\text{0}}^{\text{2}}}[2A_{\text{p}}A_{\text{p}}^{\text{*}}A_{\text{s}}e^{-2\alpha_{\text{p}}z}+A_{\text{s}}A_{\text{s}}A_{\text{s}}^{\text{*}}e^{-2\alpha_{\text{s}}z}\\ \;+2A_{\text{s}}A_{\text{i}}A_{\text{i}}^{\text{*}}e^{-2\alpha_{\text{i}}z}+4I_{\text{dc}}^{2}A_{\text{s}}\\ \;+4I_{\text{dc}}A_{\text{p}}A_{\text{i}}^{\text{*}}e^{(-\alpha_{\text{p}}+\alpha_{\text{s}}-\alpha_{\text{i}})z}e^{-{\text{Δ}}_{\beta 3}z}]\\ \gamma_{\text{i}}\frac{\partial A_{\text{i}}(z)}{\partial z}=\frac{\beta_{i}^{\text{2}}}{8I_{\text{0}}^{\text{2}}}[2A_{\text{p}}A_{\text{p}}^{\text{*}}A_{\text{i}}e^{-2\alpha_{\text{p}}z}+2A_{\text{s}}A_{\text{s}}^{\text{*}}A_{\text{i}}e^{-2\alpha_{\text{s}}z}\\ \;+A_{\text{i}}A_{\text{i}}A_{\text{i}}^{\text{*}}e^{-2\alpha_{\text{i}}z}+4I_{\text{dc}}^{2}A_{\text{s}}\\ \;+4I_{\text{dc}}A_{\text{p}}A_{\text{s}}^{\text{*}}e^{(-\alpha_{\text{p}}-\alpha_{\text{s}}+\alpha_{\text{i}})z}e^{-{\text{Δ}}_{\beta 3}z}] \end{array}\;(4)$$

where ∆ _*β*3_ = *β*_p_ − *β*_s_ − *β*_i_ and *I*_dc_ the DC-biased current. Similarly, the total phase mismatch can be obtained via ∆ _*ϕ*_ = *ϕ*_p_ − *ϕ*_s_ − *ϕ*_i_ + ∆ _*β*3_ *→* 0.

As can be seen from the frameworks described above, apart from the initial conditions e.g., the initial wave amplitudes and frequencies etc., the only unknown in the coupled mode equations is the frequency dependent complex propagation constant of the transmission line *γ*. This is the only parameter that relates directly to the physical superconducting transmission line (STL) itself (including geometries, substrates, dielectric layers etc.), and it captures all the information required to probe the evolution of the various tones propagating along the transmission length ( *z*), including all the loss mechanisms via *ℜ*( *γ*) = *α* and the frequency dependent dispersion relation through *ℑ*( *γ*) = *β*. The propagation constant *γ* can be obtained theoretically using a reduced Kirchhoff equivalent circuit of a unit cell in the form of ABCD matrixes ^24, 36^ or utilising the RLGC (Resistence-Inductance-Conductance-Capacitance) model ^37, 42^ via the series resistance, series inductance, shunt conductance and shunt capacitance calculated using complex superconducting surface impedance, and relating these quantities to *γ*. The behaviour of the full KITWPA model is then simulated assuming a translation symmetry relation (via Floquet Theorem) by multiplying a number of repeated unit cells to form the long STL.

However, this approach neglects many subtle effects such as fringing fields and discontinuities that could arise from cascading hundreds of these unit cells and it can only be used for very simple transmission line geometries e.g., it would be complicated to simulate an actual transmission line such as a coplanar waveguide (CPW) with capacitive stubs or an inverted CPW realistically. Furthermore, the calculation is lengthy with various special assumptions required for different types of materials and geometries. Apart from this frequency domain approach, another way to simulate the KITWPA performance is via the finite-difference-time-domain (FDTD) technique, but FDTD solvers are time consuming with demanding computational requirements ^24, 36^. Similar to the frequency domain approach, FDTD also suffers from the inflexibility to incorporate various different types of transmission line model.

In order to circumvent these downsides, we could take advantage of the versatility of commercial circuit simulators such as the 3D *em* software packages Ansys High Frequency Structure Simulator ^®^(HFSS), the 2.5-D Sonnet ^®^ package or Keysight’s Advanced Design System ^®^ (ADS), to fully describe the physical layout of the unit cell. This approach is extremely flexible for obtaining *γ* to feed into the framework above for calculating the gain-bandwidth product of the entire KITWPA, since one could include almost any possible structures within the modelling environment. In our case, we focus on the use of Ansys software suites ( Ansys ^®^Electronics Desktop) which make use of the full Maxwell equations and finite element method to calculate the electromagnetic properties of a 3D structure, and we use the example of a periodically loaded KITWPA for explaining the simulation procedure below. Due to the complexity of Ansys ^®^Electronics Desktop, there are no freely available alternative open-source software that can replace the full functionality of this *em* software to perform the same simulation (to our knowledge). Nevertheless, we have described the primary methodology behind the software and have provided the associated output data and analysis code. Please see *Software availability* for further information.

**Figure 1.**
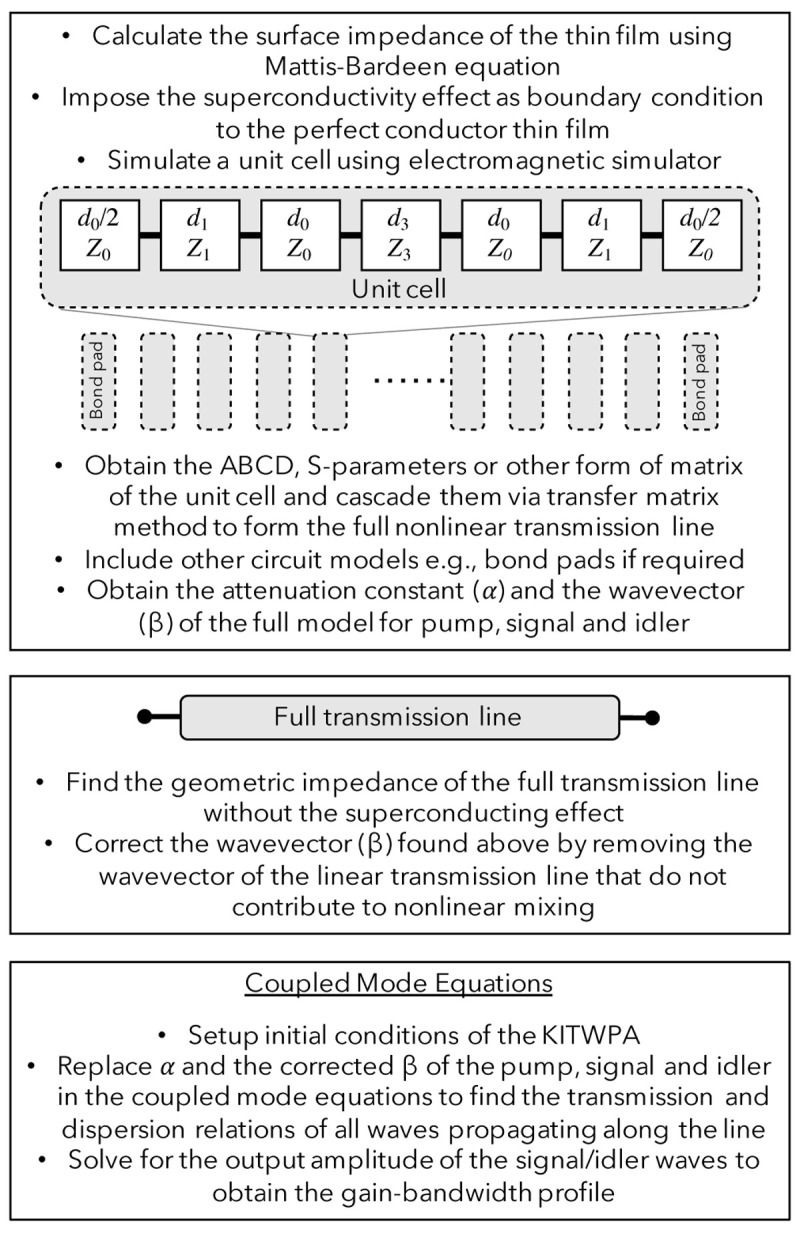
The sequence of computation procedures used to simulate the behaviour of the KITWPAs (Kinetic Inductance Travelling Wave Parametric Amplifiers), where *d*_0,1,3_ and *Z*_0,1,3_ is the length and characteristic impedance of the primary transmission line section, the first loading section and the third loading section, respectively.

To emulate the actual behaviour of the thin film in the superconducting state in the *em* model, we first calculate the complex surface impedance of the thin film using the Mattis-Bardeen equation ^43^, taking into account the film’s thickness, critical transition temperature ( *T*_c_), gap voltage ( *V*_gap_), normal resistivity ( *ρ*_N_) and the operational bath temperature ( *T*_bath_). We then assign an impedance boundary condition to a perfect conductor (PEC) modelled as the thin film, with the calculated frequency-dependent complex surface impedance values. We further include the substrate and dielectric characteristic e.g., the finite resistivity of substrate and dielectric loss tangent etc. in the *em* model as well as the more realistic topology of the KITWPA layout such as subtle effect of fringing field, radiative losses, discontinuities, geometric inductance and capacitance of the STL and many other *em* effects. Once the unit cell comprising the main transmission line and the loading sections is modelled, we cascade them using the transfer matrix approach via the dot product of the unit cell’s ABCD matrix to finally obtain *γ* of the full KITWPA chip, as shown in Figure 1. Here, we can further improve the model accuracy by including the impedance transformer and the bonding pads at the two ends of the amplifier chip. The obtained complex propagation constant is then injected into either Equation 2 or Equation 3 to probe the propagation behaviour of pump, signal and idler tones.

One distinct advantage of this methodology is that we combine the fast computation capability of the coupled mode equations with the commercial *em* software packages’s ability to accurately capture the *em* behaviour of the KITWPA, therefore better predicts the performance of the amplifier. This method is powerful as it allows us to capture the true behaviour of the STLs, including small *em* effects which could become significant when designing a full KITWPA comprising hundreds of repeated unit cells. The flexibility offered by the *em* software also provides us with a fast and simple way to explore and analyse the behaviour of different variants of KITWPA, hence designing a bespoke KITWPA with optimal performance that suits to a particular application. It also offers fast, simple and easy way to explore more complex schemes such as the use of multi-layer superconductors ^44^ for future improvement of KITWPA, open up the possibility to include more complicated designs such as the incorporation of other circuit elements apart from the unit cell like filters, and potentially other functionality apart from amplification such as frequency converters ^45^.

It is important to note that unlike the conventional theoretical methods which assume the kinetic inductance of the thin film is dominating in the case of KITWPA and hence the geometric inductance ( *L*_geo_) of the STL is neglected in the calculation, our approach takes into account all the *em* behaviour of the STL, especially the geometric inductance that do not contribute to the nonlinear mixing term described by *L*_0_. Therefore, it is imperative to correct the wave vector *β* by removing the contribution from the *L*_geo_, as shown in the second box of Figure 1.

## 3 Thin film characteristics

For the successful operation of a KITWPA, it is important to have a superconducting thin film which has the characteristic of high kinetic (surface) inductance ( *L*_s_), since the parametric gain is directly proportional to the inductance of the transmission line. This can be achieved by using high resistivity films such as Titanium (Ti), Niobium Titanium Nitride (NbTiN), or Niobium Nitride (NbN). However, it is commonly believed that high resistivity film would unavoidably incur high surface resistance ( *R*_s_) which could induce higher transmission loss, and potentially degrade the noise performance of the amplifier ^46^, preventing it from achieving the quantum limit. In this section, we explore the behaviour of several commonly used Bardeen–Cooper–Schrieffer (BCS) superconducting thin films to investigate if there is a potential solution for achieving high surface inductance (hence high gain) while keeping the surface resistance level under control (hence minimising transmission loss and added noise). As presented in Section 2, we calculate *L*_s_ and *R*_s_ using the Mattis-Bardeen equation. Apart from the three films stated above, we include low gap superconducting Aluminium (Al) and Niobium (Nb) for comparison, and Titanium Nitride (TiN) as the film’s properties such as its gap voltage can be altered by controlling the Nitrogen flow rate during film deposition. Although our studies focus mainly on the application to KITWPA, the same analysis can be useful to other applications requiring high kinetic inductance with low resistive loss films as well, such as MKIDs. In the following analysis, we assume a KITWPA operating around 10 GHz at 10 mK.

**Figure 2.**
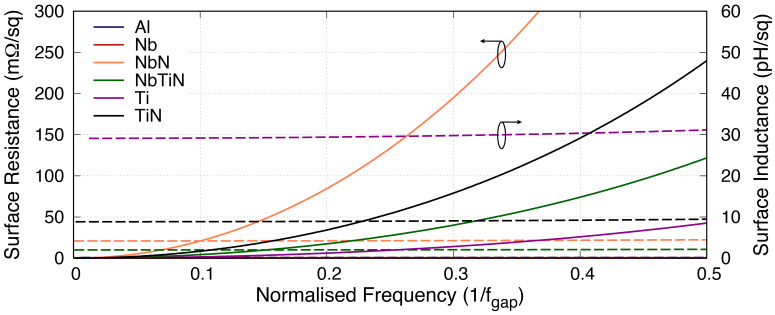
Plot showing the changes in surface impedance with relation to frequency for various 50nm thick BCS (Bardeen–Cooper–Schrieffer) films, where *f*_gap_ is the gap frequency. Solid lines represent the surface resistance, while the dashed lines represent the surface inductance. It is clear that the surface inductance is only weakly frequency dependent well below the gap frequency, but the surface resistance increases quadratically with frequency. Al, Aluminium; Nb, Niobium; NbN, Niobium Nitride; NbTiN, Niobium Titanium Nitride; Ti, Titanium; TiN, Titanium Nitride.

Table 1 shows the various superconducting materials and their related properties under investigation in this paper, and Figure 2 shows the frequency dependency relation of the surface resistance *R*_s_ and the surface inductance *L*_s_ of various 50nm thick films under study. It is obvious from the plot that *R*_s_ increases quadratically with frequency well below the gap frequency ( *f*_gap_), while *L*_s_ remains considerably constant across the same range. This is important as different material exhibits different rate of increase in *R*_s_ which is directly related to the resistivity *ρ*_N_. For example, NbN is shown to have much higher resistive loss with lower *L*_s_ compared to Ti at the same fractional frequency. From the plot, it clearly indicates that Ti would be the favourable film for KITWPA as it has the highest *L*_s_ and lowest *R*_s_ value (apart from Al and Nb). However, this could be misleading as the gap frequency of Ti under study here is approximately 41 GHz, whereas NbN has a gap frequency almost two order of magnitude higher. In this case, if we assume a KITWPA operating in the vicinity of 10 GHz, Ti film will be operating at 0.24× *f*_gap_ while NbN closer to 0.01× *f*_gap_. Referring back to Figure 2, one can immediately see that the *R*_s_ value for NbN is in fact much lower than Ti at 0.01× *f*_gap_, whilst the *L*_s_ remains at a similar level. Therefore, the choice of superconducting material used should take into account the operational frequency band of the KITWPA, not simply pursuing high *L*_s_ and low *R*_s_ film.

**Table 1.** Critical parameters of various generic BCS (Bardeen–Cooper–Schrieffer) superconducting thin films under investigation, where *T*_c_ is the critical transition temperature, *V*_gap_ the gap voltage, *f*_gap_ is the gap frequency, *f*_frac_ is the fractional frequency ( *f/f*_gap_) and *ρ*_N_ is the normal state resistivity. Al, Aluminium; Nb, Niobium; NbN, Niobium Nitride; NbTiN, Niobium Titanium Nitride; Ti, Titanium; TiN, Titanium Nitride.

Film	*T*_c_ (K)	*V*_gap_ (mV)	*f*_gap_ (GHz)	*f*_frac_ (at 10 GHz)	*ρ*_N_ (μΩcm)	Ref.
Al	1.228	0.37	89	0.11	0.63	47
Nb	9.2	2.8	677	0.014	2.85	48
NbN	14.63	4.44	1,073	0.01	220	49
NbTiN	14	4.25	1,027	0.01	100	7
Ti	0.588	0.17	41	0.24	59	44
TiN	4.39	1.33	321	0.03	140	50

**Figure 3.**
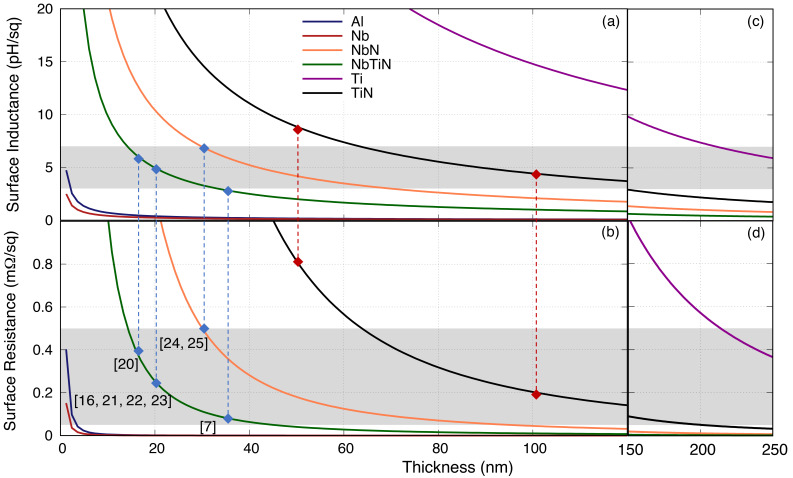
( **a**) Surface resistance and ( **b**) inductance of various thin films with different film thickness at 10 GHz, where the dots represent the values reported in the literature. Note that for data point [6] and [7] in the plot, the film used is actually NbTiN but their reported *ρ*_N_ and *V*_gap_ are closer to the value for NbN used in this paper. The two red lines represent the film under consideration for our own works. The grey boxes indicate the range of surface resistances (0.05 *< R*_s_ *<*0.5 mΩ */*□) and inductances (3 *< L*_s_ *<*7 pH */*□) where the KITWPA (Kinetic Inductance Travelling Wave Parametric Amplifiers) samples reported in the literature shows successful operation. ( **c**)–( **d**) Extending the film thickness to 250 nm. Al, Aluminium; Nb, Niobium; NbN, Niobium Nitride; NbTiN, Niobium Titanium Nitride; Ti, Titanium; TiN, Titanium Nitride.

Similarly, Figure 3 shows the behaviour of *R*_s_ and *L*_s_ with the film thickness at 10 GHz. The references shown in the plot refer to the film thickness reported in the literature that have shown to have successful operation of parametric amplification. Using this information, we have charted out an area of *R*_s_ and *L*_s_ that has shown to be working well for KITWPA, as the baselines for this study. Note that strictly speaking the *R*_s_ curves indicate the upper limit for the thickness used of a certain film, while the *L*_s_ curves indicate the lower limit, since as mentioned earlier, films with higher *L*_s_ and lower *R*_s_ are preferred here.

In the first instance, Figure 3 seems to suggest that only three films fall within the grey area regime i.e., the NbTiN, NbN and TiN. However, as the *R*_s_ and *L*_s_ is inversely proportional to the film thickness, one notes immediately that in fact the Ti film could also work if the film is much thicker than 220 nm, while the non-conventional Al and Nb would also work albeit with an unreasonably thin film ( *<*2 nm). This finding is interesting, as it indicates that as long as we can control the film thickness, all of these films (or in fact any superconducting films) will be feasible for developing a functional KITWPA. Again, this shows that the intrinsic properties of the film are not as strictly important as one may have thought, as long as other film’s properties such as thickness can be adjusted to provide the required *L*_s_ and minimal *R*_s_ to achieve high gain and low loss parametric amplification process.

**Figure 4.**
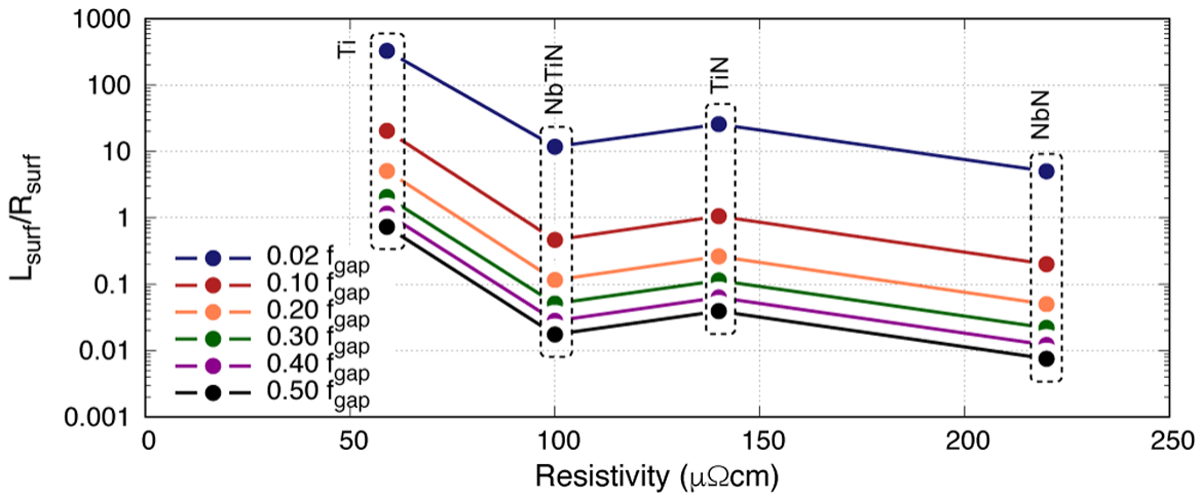
Plot showing the ratio of *L*_s_/ *R*_s_ (surface inductance/surface resistance) for 50nm thick Ti (Titanium), NbTiN (Niobium Titanium Nitride), TiN (Titanium Nitride) and NbN (Niobium Nitride) at different normalised frequencies, where *f*_gap_ the gap frequency.

Since we assume that a good thin film for a KITWPA would have high *L*_s_ and low *R*_s_, it is imperative to find the *L*_s_ */R*s ratio for different films. Figure 4 shows this ratio for four thin films under study at 50nm thick at various operating frequencies ^1^. The first information gathered from this plot is that the higher the operating frequency, the lower the ratio is, suggesting that for high frequency KITWPA operation one would require high gap superconductor as expected. Secondly, there is no clear trend of the ratio with resistivity *ρ*_N_. Once again, this plot appears to imply that Ti is a relatively good film to use compared to others since it has almost an order of magnitude higher *L*_s_/ *R*_s_ ratio than other films. However, as noted earlier, this comes with the price of extremely low frequency operation due to the low gap frequency limit. Again, at *f*_op_ → 10 GHz, the *L*_s_/ *R*_s_ ratio for Ti would be at a similar range as NbN. Therefore the use of *L*_s_/ *R*_s_ ratio to find the optimal film characteristic may leads to unexpected results that is not indicative to the actual performance of the KITWPA.

**Figure 5.**
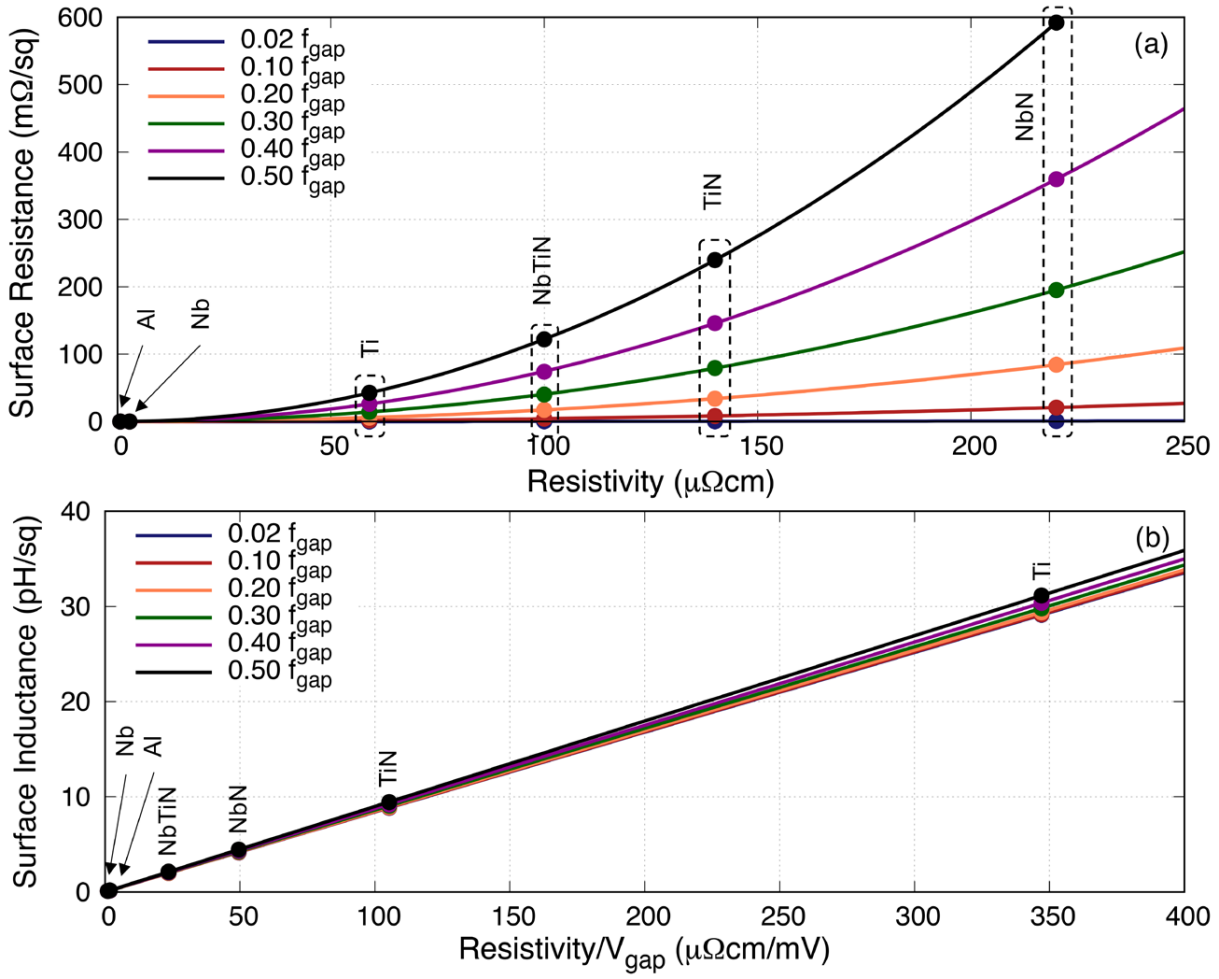
Plots showing the relation between ( **a**) surface resistance *R*_s_ with resistivity *ρ*_N_ and ( **b**) surface inductance *L*_s_ with the ratio of *ρ*_N_/ *V*_gap_ of various 50nm films at different fractional frequency, where *f*_gap_ the gap frequency. Ti, Titanium; NbTiN, Niobium Titanium Nitride; NbN, Niobium Nitride; Al, Aluminium; Nb, Niobium; TiN, Titanium Nitride.

Although the above plots have provided good information regarding how *R*_s_ and *L*_s_ change with frequency and film thickness, it is inconclusive which materials or their thickness are optimal for designing a KITWPA i.e., highest *L*_s_ with lowest *R*_s_. In Figure 5 we relate the two parameters of interest with the resistivity *ρ*_N_ and the gap voltage *V*_gap_ of various 50nm films. As expected, higher *ρ*_N_ would induce higher *R*_s_. However, rather unexpectedly, *L*_s_ displays no clear trend with either *ρ*_N_ or *V*_gap_, but in fact is linearly proportional to the ratio of *ρ*_N_/ *V*_gap_. Furthermore, *R*_s_ increases significantly with frequency but *L*_s_ stays rather constant, just as indicated earlier. These two plots imply that one could in fact control the surface inductance and resistance of the film independently by engineering the film’s resistivity and gap voltage. For example, to achieve lower *R*_s_ one could lower *ρ*_N_, and in order to retain the same *L*_s_ value, we reduce *V*_gap_ to compensate for the decrease of *L*_s_ due to lower *ρ*_N_. This is doable since *V*_gap_ is not a function of film thickness, therefore we can alter the thickness *R*_s_, once we have engineered the film to have a lower *V*_gap_. This solution should be tangible for higher gap superconductor such as TiN, NbTiN and NbN where the operational frequency is well below the gap frequency.

### 3.1 Engineered TiN thin films

In order to test the above postulate, we have fabricated several batches of TiN films with different Nitrogen flow rates to alter the gap voltage (hence the gap frequency).

**Figure 6.**
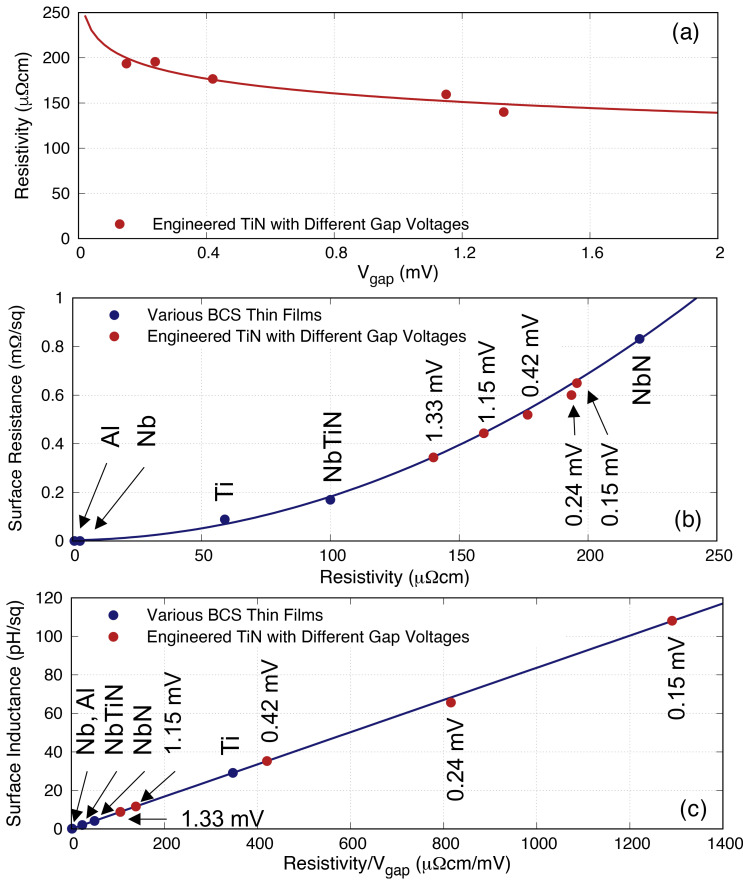
( **a**) Plot showing the resistivity of various engineered TiN film with different gap voltages, where *V*_gap_ is the gap voltage. ( **b**) Relation between surface resistance and resistivity at 0.02 *f*_gap_ for various BCS (Bardeen–Cooper–Schrieffer) thin films under study, along with the engineered TiN films, all at 50nm thickness. & ( **c**) Similar to ( **b**) for the relation between surface inductance and the ratio of *ρ*_N_/ *V*_gap_. The various engineered TiN films are labelled with their corresponding *V*_gap_. Ti, Titanium; NbTiN, Niobium Titanium Nitride; NbN, Niobium Nitride; Al, Aluminium; Nb, Niobium; TiN, Titanium Nitride.

We then measured the resistivity of the film ^2^ to find the relation between *V*_gap_ and *ρ*_N_. The result is depicted in Figure 6 (a), showing that *ρ*_N_ decreases gradually with *V*_gap_, but not significantly. In other words, it may seem feasible to assume that *ρ*_N_ stays rather constant with large changes in *V*_gap_ e.g., *ρ*_N_ drops only by about 1/3 of its value when *V*_gap_ is increased from 0.15→1.33mV, almost an order of magnitude higher. This means that although *ρ*_N_ will increase slightly (hence higher resistive loss) when *V*_gap_ is reduced, *L*_s_ would increase significantly, hence we can achieve higher *L*_s_ while retaining similar level of *R*_s_ to produce a KITWPA with much shorter transmission length.

Figure 6 (b) & (c) reproduce the 0.02 *f*_gap_ curve shown in Figure 5 (a) & (b) but with added points of these engineered TiN films. As can be seen, indeed we can achieve higher *L*_s_ with the engineered films, while retaining the *R*_s_ within the same range. For example, the TiN film with *V*_gap_ = 0.42mV is clearly shown to have a lower *R*_s_ at 0.52 mΩ */*□ but *L*_s_ is now much higher, close to 35 pH */*□, compared to NbN with *R*_s_ = 0.83 kΩ */*□ and *L*_s_ = 4.2 pH */*□. This suggests that our earlier proposed solution may work to achieve higher *L*_s_ and lower *R*_s_ by engineering *ρ*_N_ and *V*_gap_ of the film.

**Figure 7.**
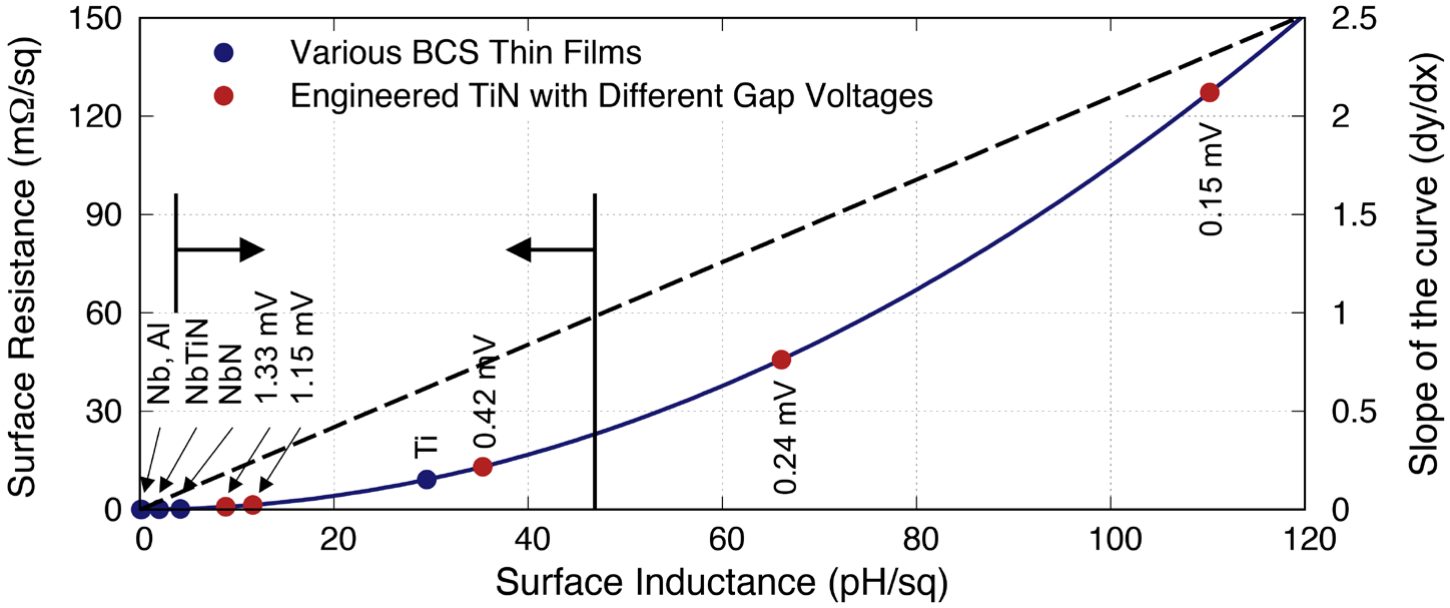
The relation between surface resistance and inductance at 10 GHz for various BCS (Bardeen–Cooper–Schrieffer) thin films under study, along with the engineered TiN films, all at 50nm thickness. The graph shows a clear quadratic relation between *R _s_* (surface resistance) and *L _s_* (surface inductance). The dashed line plots the derivative of the quadratic relation, where the arrows show the possible operational range for KITWPA (Kinetic Inductance Travelling Wave Parametric Amplifier). The lower limit was taken from Figure 3 where *L _s_* = 3 pH/□, while the upper limit indicates the point where the slope of the quadratic curve is less than 1. Al, Aluminium; Nb, Niobium; NbN, Niobium Nitride; NbTiN, Niobium Titanium Nitride; Ti, Titanium; TiN, Titanium Nitride.

However, it is obvious that lowering *V*_gap_ would affect the frequency range in which the KITWPA can operate, as well as the bath temperature. Therefore, it would be indicative to perform the same analysis at a fixed operational frequency instead of fractional frequency. Figure 7 shows the relation between *R*_s_ and *L*_s_ for all the 50nm film under study here, including the engineered TiN films, which surprisingly show a clear quadratic relation between the two parameters, and it is consistence regardless of the film types i.e., independent of *ρ*_N_ & *V*_gap_. This is in stark contrast with our assessment earlier, as this relation shows that the film properties have no significant effect on altering the *R*_s_- *L*_s_ relation. In another words, the ideal films would be located near the bottom right of the graph, but clearly from Figure 7 this would be impossible. The reason behind this is because lowering *V*_gap_ inevitably increases the normalised frequency (1/ *f*_gap_), which in turn increases *R*_s_ as shown earlier in Figure 2.

**Figure 8.**
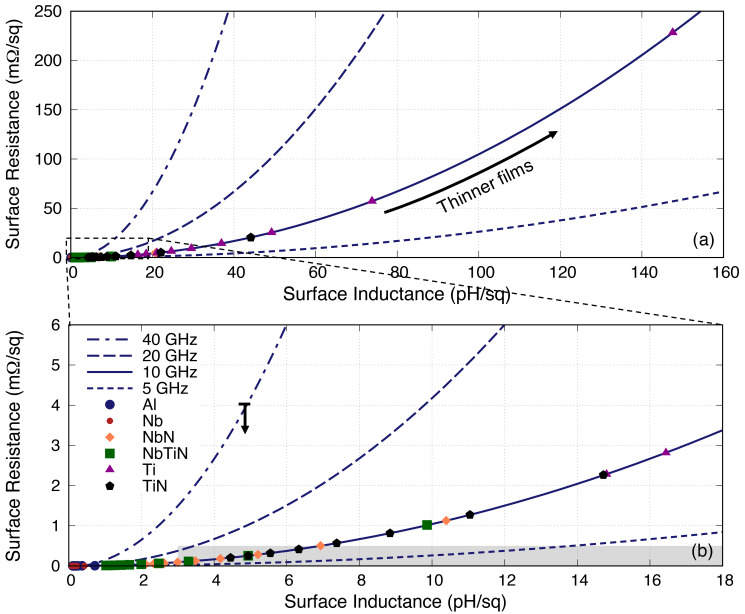
Plot showing the clear quadratic relation between surface inductance and surface resistance of different films with different thicknesses at different operating frequencies (data for TiN shown here refers to the TiN with *V*_gap_ = 1.33 mV). The surface impedances were calculated from 10nm up to 100nm with 10nm interval shown in dots. The grey box indicates the lower limit of the surface inductance required with the upper limit dictated by the surface resistance before the film gets too lossy (reference to the grey boxed in Figure 3). The thin films that fall in to the greyed out regime is NbTiN with *t ≈* 15–35 nm, NbN with *t ≈* 30–70 nm, TiN with *t ≈* 65–150 nm & Ti with *t >* 220 nm. Al, Aluminium; Nb, Niobium; NbN, Niobium Nitride; NbTiN, Niobium Titanium Nitride; Ti, Titanium; TiN, Titanium Nitride.

Another interesting observation here is that the quadratic nature of the relation also indicates that the surface resistance increases faster than the inductance when *dR*_s_ */dL*_s_ *>* 1. This could indicate that for a successful KITWPA operation at 10 GHz, the thin film used should have an *R*_s_ *<* 23 k *Ω/*□ with *L*_s_ *>* 3 pH */*□ as shown by the arrows in Figure 7. Figure 8 plots the same *R*_s_- *L*_s_ relation for all films with different thickness at different operating frequencies, again showing the same quadratic relation between the two parameters regardless of the film thickness and operating frequency as well. Here, we exclude the engineered TiN films as it makes the plot too crowded to view, but all the points would lie exactly on the 10 GHz curve ^3^. Obviously for KITWPA the favourable scenario is high *L*_s_ with low *R*_s_ as previously stated. However, this relation implies that higher *L*_s_ would induce higher *R*_s_ regardless of the film’s properties, hence it is implausible to reduce the *R*_s_ without sacrificing for lower *L*_s_ value.

As indicated earlier, the surface resistance increases faster than the inductance above *dR*_s_ */dL*_s_ = 1, and the situation is worse with higher frequency where the rate of increase (i.e., the derivative of the curve) becomes steeper, in contrast to the low frequency operation where the relation is almost linear. This unfortunately suggests that KITWPA may not work well at high frequencies (e.g., at millimetre-wave regime) even with high gap superconductors, which again was actually already hinted at Figure 2. One may argue that for high frequency operation, one could choose the regime with lower inductance where the *R*_s_- *L*_s_ relation is more linear. But this may not lift the predicament because the film needs to have a certain lower limit value of *L*_s_ as indicated by the grey box. A clear example is shown by the 40 GHz curve where it does not intersect with the grey box at all. However, it is worthwhile noting that these limits are not fundamental limits but simply the indicative reference points from the working devices reported in the literature. In particular, results reported in 25 showed that their KITWPA could work up to 40 GHz with their NbTiN film (which have closer properties to the NbN parameters used here). Therefore, this may imply that one could in fact tolerate higher resistive loss up until 4 k *Ω/*□ as shown by the down arrow in Figure 8 (b).

It is interesting to note that the relation between *R*_s_ and *L*_s_ shown in Figure 6 and Figure 8 is universal and independent of the properties of the thin film used. This may seems counterintuitive, but it is not hard to prove mathematically. From Figure 5, it is clear that *L*_s_ ∝ *ρ*_N_/ *V*_gap_ and *R*_s_ ∝ $\rho_{\text{N}}^{2}.$ By rearranging these two equations, we arrive at *R*_s_ ∝ ( *hf*_op_ *L*_s_/ *Ne*) ^2^, where *V*_gap_ = *hf*_gap_ */e*, *f*_op_ is the operational frequency, *N* = *f*_op_/ *f*_gap_ = *V*_op_/ *V*_gap_, and *e* & *h* the electron charge and Planck constant. Therefore, the relation between the *R*_s_ and *L*_s_ is in quadratic nature, with ( *hf*_op_ */Ne*) ^2^ as the coefficient, as shown in Figure 6 and Figure 8, and that the slope of the curve scales with frequency. Similarly, the surface resistance is also related to the operating frequency in quadratic fashion with ( *hL _s_/Ne*) ^2^ as the coefficient, and as shown in Figure 2, where higher *L*_s_ (or *ρ*_N_/ *V*_gap_) incurs higher *R*_s_.

These findings are in disagreement to conventional presumption that film with high resistivity or *V _gap_* is required for KITWPA operation. In fact, it indicates any superconducting thin films could work as long as the correct thickness is used (below the penetration depth but not too thin to incur too high resistive loss), and that even film such as superconducting Aluminium or Niobium could be used to fabricate KITWPA, albeit with unreasonably thin films.

Therefore, we identify that for our design, we shall focus on the use of 100nm TiN film with *V*_gap_ = 1.33mV ( *T*_c_ = 4.39 K) as it has similar *R*_s_ and *L*_s_ values as most of the successful examples shown in Figure 3. For the following analyses, we further include another option with half the TiN film thickness to show the impact of higher *R*_s_ and *L*_s_ on the performance of the KITWPA.

## 4 Substrate and topology considerations

**Figure 9.**
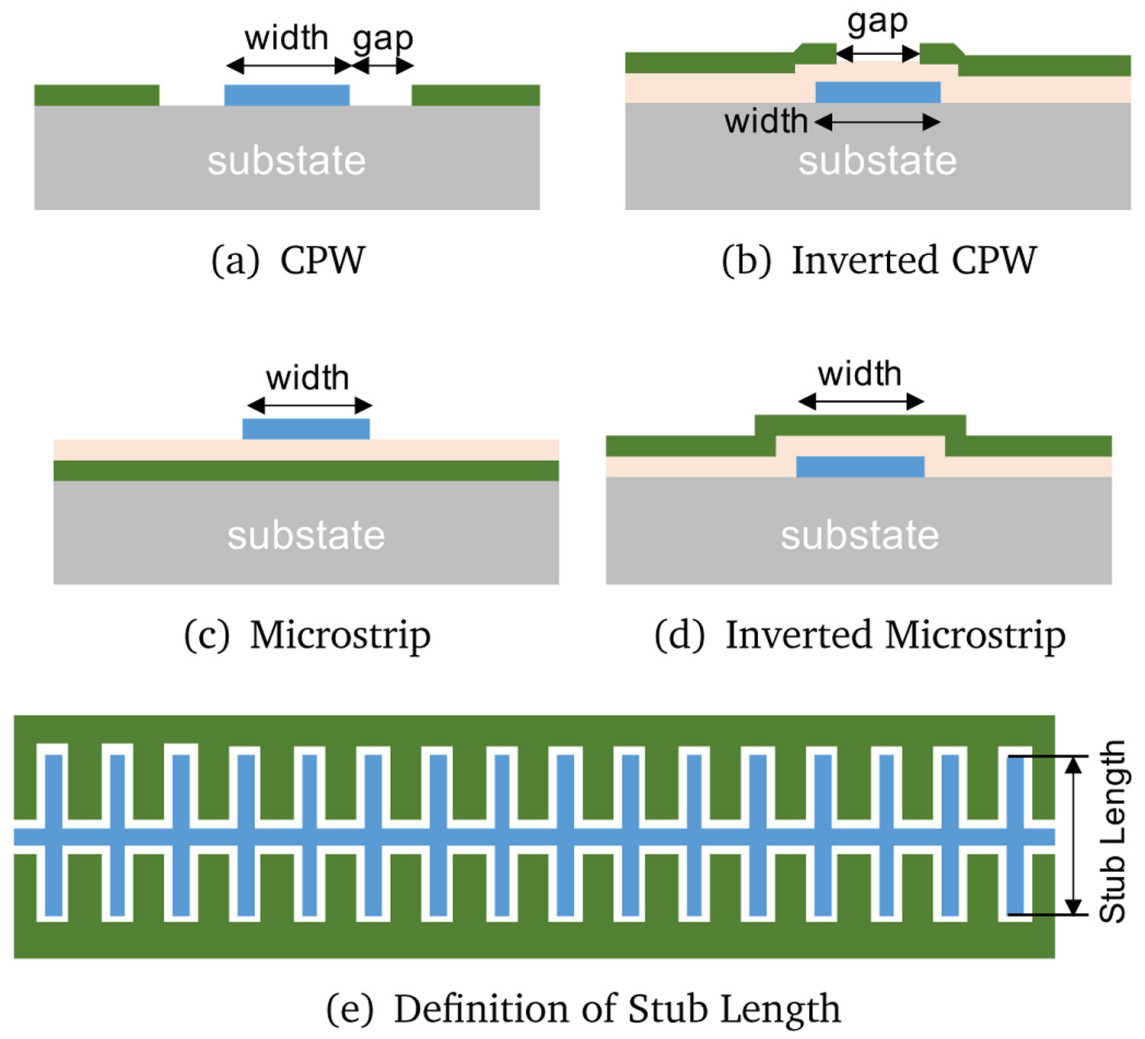
Various types of transmission lines under investigation, where CPW stands for coplanar waveguide. The ground layers are coloured green, dielectric layer light orange and the wiring layer blue. For all the analysis presented, we assume a substrate thickness of 500 µm for all topologies and substrate choice.

The choice of the superconducting thin film forms only part of the design consideration for developing an optimal KITWPA. The planar circuit amplifier can in principle be formed using any type of transmission line topology fabricated on different varieties of supporting substrate. In this section, we explore how these transmission line topology and substrate choices would affect the performance of the KITWPA. For this exercise, we too make use of *em* simulators Ansys HFSS and the Mattis-Bardeen equations. The source code for calculating the superconducting surface impedance via the Mattis-Bardeen equation, namely the SuperMix software libraries, were previously developed by several contributors at the California Institute of Technology (CalTech), and not by the authors of this article. Source code of SuperMix is available at: here In particular, we focus on the conduction and dielectric losses, the compactness of the device and the feasibility of fabrication in terms of circuit dimensions. Figure 9 shows the four different types of STL topologies we investigate here i.e., coplanar waveguide (CPW), inverted CPW, microstrip, and inverted microstrip line.

Among the chosen four topologies, the inverted lines have the advantage of protecting the long transmission line via the top ground layer, especially the central conductor or the wiring layer, from potential physical damage during handling of the device such as subsequent chip dicing and wire bonding process after fabrication. This also helps to avoid potential unwanted scratches, which could cause open circuit or debris of conducting materials that cause short circuit to ground. However, the downside of such protective layer is the need for a dielectric layer, which means that the wiring layer is now sandwiched between two non-conductive layers i.e., the thin dielectric layer and the supporting substrate, hence could potentially increase the dielectric losses and induce higher noise due to the two level system (TLS) noise mechanism. On the other hand, conventional CPW and microstrip only have a single contact interface between the wiring layer and the dielectric or the substrate, but it may be prone to mechanical damage as the transmission line is now completely exposed.

As we have shown that the choice of superconducting thin film is relatively unimportant as long as the thickness of the film can be controlled accurately, for all the following analysis, we shall only focus on the TiN film with a *T*_c_ = 4.39 K. We include both 50nm and 100nm options to demonstrate the effect of the relative surface resistance and inductance. For all the STL topologies under study here, we assume that the dielectric layer is formed using silicon monoxide (SiO) with a resistivity *ρ* = *∞*, dielectric loss tangent tan *δ* = 0.004 and relative permittivity *ε*_r_ =5.8. We explore three different types of commonly used substrate i.e., high resistivity silicon, sapphire and quartz (the characteristics of these substrates are listed in the footnote of Table 2), all with the same thickness of 500 µm. All the transmission line dimensions are optimised to achieve a characteristic impedance *Z*_0_ of 50Ω to match the impedance of the external circuitry. Due to this requirement, we exclude the choice of generic CPW because the typical dimensions used for this type of STL would result in a much higher *Z*_0_ because of the high surface kinetic inductance ( *Z*_0_ ∝ $\sqrt{L/C}$ ) of the film ^4^. Instead, we relax the need for an unrealistically small gap (increase *C*) or large central strip width (to reduce the geometric *L*), by introducing a series of capacitive stubs along the CPW as shown in Figure 9 (e) to significantly increase the shunt capacitance while retaining a similar value of surface inductance ^22, 33^.

**Table 2.** Electromagnetic characteristics of different types of transmission lines on different substrates for a 50nm thick TiN with *T*_c_ = 4.39 K. Here we assume an SiO (silicon monoxide) thickness of 100 nm. CPW, coplanar waveguide; TiN, Titanium Nitride.

	Silicon ^1^			
	CPW	Inv. CPW	Microstrip	Inv. M-strip
Width	10 μm	5 μm	2.1 μm	2.0 μm
Gap	5 μm	4.65 μm	NA	NA
*L*_stub_	72 μm	NA	NA	NA
*L*_*λ*/2_^5^	910 μm (30 stubs)	750 μm	350 μm	330 μm
S _21_:10 GHz ^4^	–1.10 dB	–2.88 dB	–3.87 dB	–3.59 dB
S _21_:30 GHz ^4^	–4.27 dB	–12.89 dB	–16.84 dB	–15.81 dB
	Sapphire ^2^			
	CPW	Inv. CPW	Microstrip	Inv. M-strip
Width	10 μm	5.05 μm	2.1 μm	1.95 μm
Gap	5 μm	4.65 μm	NA	NA
*L*_stub_	101 μm	NA	NA	NA
*L*_*λ*/2_	830 μm (28 stubs)	785 μm	340 μm	336 μm
S _21_:10 GHz	–0.37 dB	–2.66 dB	–4.15 dB	–3.75 dB
S _21_:30 GHz	–3.33 dB	–12.61 dB	–17.90 dB	–16.72 dB
	Quartz ^3^			
	CPW	Inv. CPW	Microstrip	Inv. M-strip
Width	10 μm	5.24 μm	2.1 μm	2.03 μm
Gap	5 μm	4.65 μm	NA	NA
*L*_stub_	288 μm	NA	NA	NA
*L*_*λ*/2_	725 μm (24 stubs)	785 μm	350 μm	345 μm
S _21_:10 GHz	–0.31 dB	–3.36 dB	–3.87 dB	–3.92 dB
S _21_:30 GHz	–3.05 dB	–14.88 dB	–16.84 dB	–17.17 dB

^1^Relative dielectric constant _r_ =11.9, resistivity *ρ* =15 kΩcm, loss tangent tan *δ*=1.2×10 ^−5^. ^2^_r_ =9.4, *ρ* =∞, tan *δ*=8.0×10 ^−8^. ^3^_r_ =3.78, *ρ* =∞, tan *δ*=0. ^4^70 wavelengths at 24 GHz. ^5^At 24 GHz. ^6^ *L*_stub_ is the length of the stubs, *L*_*λ*/2_ is the half wavelength length of the section at 24 GHz and S _21_ is the insertion loss.

From Table 2 and Table 3, one immediately notes that the transmission (insertion) loss of the CPW in terms of S _21_ is much lower than the other three STLs. This is because the effective dielectric loss seen by the CPW electric field lines is in fact half of that the substrate dielectric constant, as the top half of the field is above the CPW and travels in the free space. In other words, the filling factor is 50% because the field lines are evenly divided between the free space and the substrate; whereas the inverted CPW has a majority of the fields enclosed between the top ground plane and the substrate. For microstrips and inverted microstrips, the field lines are tightly confined within the thin dielectric layer, hence the losses are inevitably higher.

**Table 3.** Electromagnetic characteristics of different types of transmission lines on different substrates for a 100nm thick TiN with *T*_c_ = 4.39 K. The SiO (silicon monoxide) thickness is increased to 200nm for easier match to *Z*_0_ = 50Ω to avoid need for narrow strip line. The symbols used in the table are given in Table 2. CPW, coplanar waveguide; TiN, Titanium Nitride.

	Silicon			
	CPW	Inv. CPW	Microstrip	Inv. M-strip
Width	10 μm	5.06 μm	2.16 μm	1.93 μm
Gap	5 μm	5 μm	NA	NA
*L*_stub_	50 μm	NA	NA	NA
*L*_*λ*/2_	1.27mm (42 stubs)	1.19mm	650 μm	600 μm
S _21_:10 GHz	–0.91 dB	–2.00 dB	–3.47 dB	–3.07 dB
S _21_:30 GHz	–2.17 dB	–7.36 dB	–13.03 dB	–11.58 dB
	Sapphire			
	CPW	Inv. CPW	Microstrip	Inv. M-strip
Width	10 μm	5.18 μm	2.16 μm	2 μm
Gap	5 μm	5 μm	NA	NA
*L*_stub_	74 μm	NA	NA	NA
*L*_*λ*/2_	1.14mm (38 stubs)	1.2mm	650 μm	610 μm
S _21_:10 GHz	–0.14 dB	–1.87 dB	–3.47 dB	–3.04 dB
S _21_:30 GHz	–1.26 dB	–7.66 dB	–13.03 dB	–11.79 dB
	Quartz			
	CPW	Inv. CPW	Microstrip	Inv. M-strip
Width	10 μm	5.48 μm	2.16 μm	2.1 μm
Gap	5 μm	5 μm	NA	NA
*L*_stub_	235 μm	NA	NA	NA
*L*_*λ*/2_	840 μm (28 stubs)	1.25mm	650 μm	625 μm
S _21_:10 GHz	–0.10 dB	–2.55 dB	–3.42 dB	–3.21 dB
S _21_:30 GHz	–0.98 dB	–9.71 dB	–12.87 dB	–12.20 dB

However, microstrip and inverted microstrip line have the benefit of shorter physical length (due to higher geometrical shunt capacitance), and hence can produce a much compact KITWPA design and potentially increase yields due to a smaller footprint area for damage. The microstrip family is also easier to design, as CPW tents to generate higher unwanted modes if not being designed properly. Therefore, for the CPW families simulated here, equipotential bridges are deployed along the line to ensure that the two ground planes are consistently in electrical contact. From the point of view of insertion loss, it is easy to conclude that inverted CPW offers no advantage compared to the other STLs as it is as lossy as the microstrip family, but have comparative long electrical length like the CPW. Comparing the microstrip with the inverted microstrip, it can be easily noted that the performances are relatively similar, but the inverted microstrip offers additional protective layer in terms of the top ground plane, with the only downside that it may induce higher TLS losses.

In terms of the substrate choices, one notes immediately from Table 2 and Table 3 that the characteristics of the microstrip family are relatively unaffected by the choice of the substrate as the field lines are concentrated in the dielectric layer. However, we can see that for case of CPW, silicon generally induces higher loss than sapphire and quartz which are ceramic in nature ( *ρ* = *∞*), whereas silicon is a semiconductor that can partially conduct current. Hence, even with a very high substrate resistivity of *ρ* =15 kΩcm, the losses are still visibly higher due to the long transmission length. Between sapphire and quartz, the latter induces the lowest loss because it has the lowest loss tangent, but the length of the capacitive stubs ( *L*_stub_) increases substantially compared to silicon and sapphire due to the low relative permittivity. The effective wavelength of both ceramic substrates also decreases compared to silicon, hence requiring fewer stubs with shorter physical STL length. Therefore, we conclude that for case of CPW, sapphire is the optimal choice as it’s less lossy than silicon and requires significantly shorter stubs than quartz, hence reducing the footprint of the amplifier chip.

Comparing the difference between using 50nm and 100nm TiN film, we note that the S _21_ loss is less with the 100nm film, as the surface resistivity is lower. However, as discussed in the earlier section, lower *R*_s_ indicates lower *L*_s_, therefore the effective wavelength is now longer (requiring more stubs). Nevertheless, the stub length is relatively short for the 100nm film, hence compensating for the higher number of stubs required, and most probably would result in a similar compactness in terms of the amplifier footprint. Note that the width of the microstrip line is similar for both 50nm and 100nm cases. In principle this should not be the case since a lower *R*_s_ from the 100nm film will decrease *Z*_0_ and hence a narrower line is needed to reduce the shunt capacitance and increase geometric inductance to achieve *Z*_0_ = 50Ω. However in our case, we opt to increase the thickness of the SiO layer from 100 nm to 200 nm, hence effectively reducing the shunt capacitance to avoid the need for much narrower line width that could be difficult to fabricate using standard photolithography techniques, especially to maintain the consistency of width for such a long transmission length. Furthermore, with narrow microstrip, the characteristic impedance becomes more sensitive to the width and hence more susceptible to the fabrication error, affecting our ability to achieve 50Ω line accurately.

In summary, the use of a thinner 50nm film results in higher losses compared to the 100nm film but requires shorter transmission length. A CPW has the lowest insertion loss among all STL topologies, especially with sapphire or quartz substrate, but the stub length for using quartz become inconveniently long, hence the use of sapphire substrate is a better option. An inverted CPW may produce more compact amplifier design as it can achieve 50Ω characteristic impedance without the need for capacitive stubs, but the loss is high, similar to the level of the microstrip family, hence offers no added advantage here. For the microstrip and the inverted microstrip, their characteristics are largely similar, and are unaffected by the choice of substrate. However inverted microstrip allows for protection of the main transmission line from potential physical damage, as well as better thermalisation as the wiring layer is now directly in contact with the substrate. Therefore, we identify that to achieve the lowest loss (hence least resistive heating) design with relatively small footprint, one should opt for using the 100nm film forming a CPW with capacitive stubs on top of sapphire. On the other hand, to achieve a more compact KITWPA design with shortest effective wavelength, we should choose inverted microstrip design with 50nm thick TiN. We shall include both designs in the subsequent sections and compare their characteristics and performances.

## 5 Designs of KITWPA

In this section, we perform the gain-bandwidth product prediction using the method presented in Section 2 for both KITWPAs identified earlier. Please note that all data underlying the graphs and the scripts for plotting the graphs are available in *Underlying data* and *Extended data* ^51^. To achieve exponential gain, the power dependent phase difference between all the propagating tones needs to be minimised. As explained previously, this can be done by altering the dispersion relation of the transmission line through periodically varying the impedance along the line i.e., the periodic loading scheme. Here, we aim to achieve more than 20 dB gain centred at 8 GHz with minimal length for compactness. For an optimal amplifier design, we take into account considerations that are needed to mitigate risk of physical damage, promoting uniformity of the transmission line, and target for minimum pump power requirement. The last requirement is important as the coupled mode equations assume *I*_p_ ≪ *I*_0_, hence at high pump power level, the gain-bandwidth prediction may not be completely reliable anymore. Furthermore, high pump power could also potentially increase amplifier noise through the resistive loss.

As explained in Section 2, for these simulations, we include all the loss mechanisms such as substrate resistivity, dielectric loss tangent etc., as well as the equipotential bridges for the case of CPW amplifiers, as the added extra capacitance from these bridges would slightly shift the propagation length as well as reducing the radiative loss.

### 5.1 Validation of design methodology

**Figure 10.**
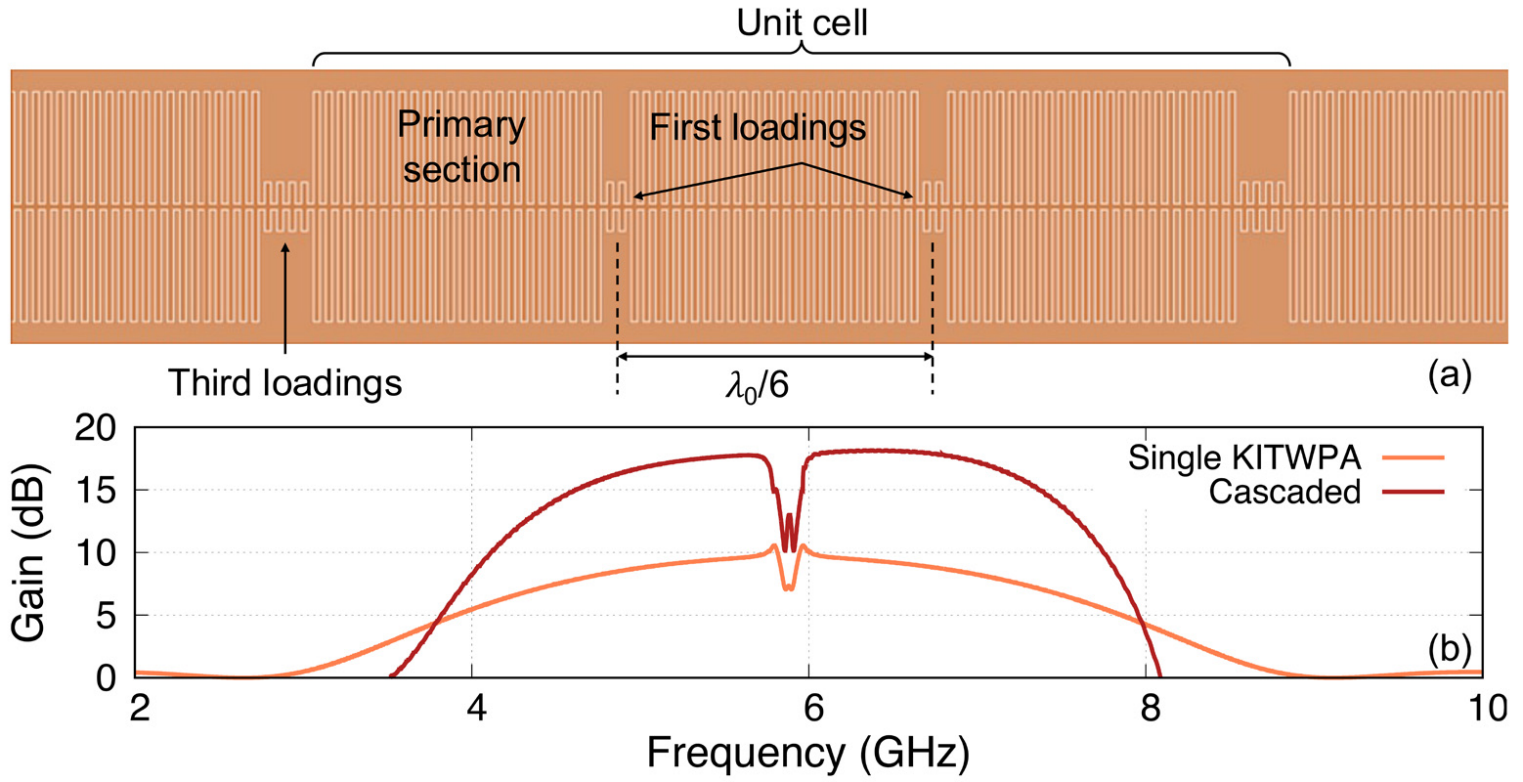
( **a**) Topology of a KITWPA (Kinetic Inductance Travelling Wave Parametric Amplifier) reported in 22, where *λ*_0_ is the central frequency wavelength. ( **b**) Simulated gain-bandwidth profiles of the amplifier using the method presented in this paper.

Before we start to simulate the KITWPA using the methodology presented in Section 2, to ensure that the proposed method is feasible, here we reproduce the gain-bandwidth product of a measured KITWPA reported by Chaudhuri *et al.* ^22^ using the information provided in the paper. The KITWPA was designed to operate at a central frequency of *∼*6 GHz and was fabricated using 20nm niobium titanium nitride (NbTiN, *T*_c_ = 15 K) film deposited on top a silicon substrate. The transmission line is configured as a coplanar waveguide (CPW) with capacitive stubs as shown in Figure 10(a) to achieve a 50Ω line. The central strip and gap width of the CPW and the stubs are 2 µm wide, and the capacitive stub length is reduced by a factor of 5 at every one-sixth of a wavelength ( *λ*_0_) at the central frequency (increasing the characteristic impedance from 50 to 111Ω) to create the stopbands at *∼*18 GHz for pump’s third harmonic suppression. The length of every third loading sections is doubled from 2 to 4 stubs to create the sub-stopbands at 6 and 12 GHz. Here, the unit cell is formed by interweaving three 50Ω sections with three loading sections, and the total electrical length of the amplifier is 140 unit cells long.

Using the method described above, we calculate the parametric gain of the KITWPA. As shown in Figure 10(b), we could reproduce the 9 dB gain of the KITWPA as claimed in 22. In the paper, they reported that they cascaded two of these linear amplifiers to achieve greater than 15 dB gain. Therefore, we further simulated the gain of the cascaded KITWPAs by taking the output signal amplitude of the first amplifier as the input to the second KITWPA. As shown in Figure 10(b), we managed to obtain the gain curve that is very close resemblance to the measured performance shown in Figure 3 of 22. Therefore, we are confident that our proposed method can accurately predicts the gain-bandwidth performance of a KITWPA.

### 5.2 CPW with 100nm TiN film

In this section, we focus on the design of the CPW parametric amplifier that is topologically similar to the example shown above but formed using 100nm TiN film on a sapphire substrate. Here we utilise a similar periodic loading scheme for phase matching and pump’s third harmonic suppression, except we alter the characteristic impedance *Z*_0_ of the third loading section by removing the capacitive stubs (instead of the loading length) to increase the difference between the third loading impedence to the primary loading impedance, and thereby produce stronger sub-stopbands for dispersion correction. The dimensions of the 50Ω main transmission line, the primary loading section and the third loading section making up the unit cell is tabulated in Table 4.

**Table 4.** Dimension of the transmission line forming a single unit cell for the CPW (coplanar waveguide) KITWPA (Kinetic Inductance Travelling Wave Parametric Amplifier) on a sapphire substrate, where *Z*_0_ is the characteristic impedance and *L*_stub_ is the length of the stubs. TiN, Titanium Nitride.

	CPW with 100nm TiN film		
	Primary Line	1 ^st^ Loading	3 ^rd^ Loading
*Z*_0_	50Ω	60Ω	90Ω
Width	10 μm	10 μm	5.7 μm
Gap	5 μm	5 μm	5 μm
*L*_stub_	74 μm	36 μm	NA
Length	34 stubs	4 stubs	200 μm

**Figure 11.**
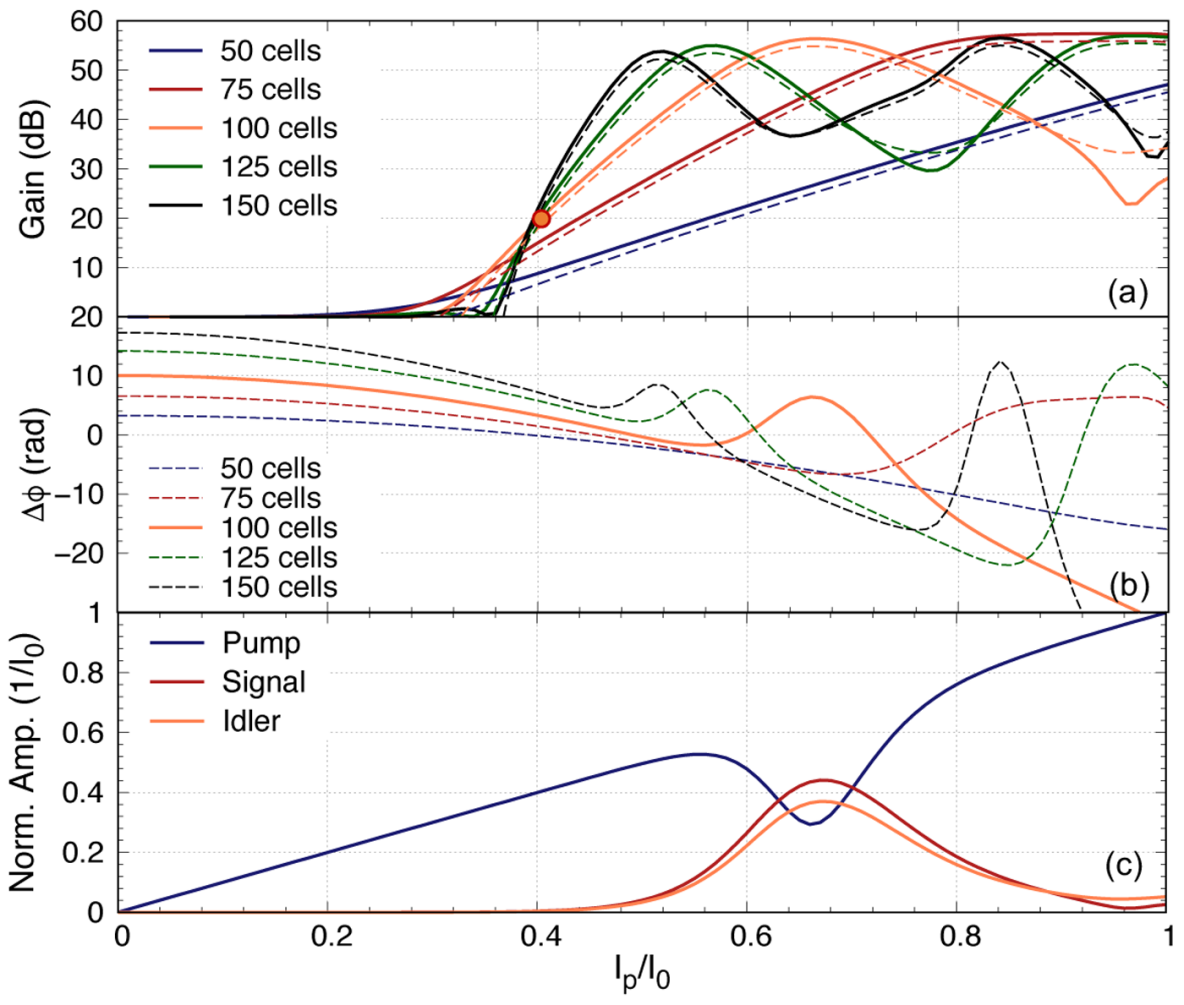
( **a**) The relation of parametric gain plotted against the pump power for different length of the amplifier, where ∆ _*ϕ*_ is the total phase mismatch and I _0_ is the critical current. The solid line represent the signal gain, while the dashed line the idler gain (reference to input signal power). ( **b**) The total phase mismatch plotted against the pump power. ( **c**) Curves showing the relation between the pump, signal and idler tones reacting to each other for the 100 cells amplifier. All curves were plotted with signal frequency of 9 GHz and pump frequency of 7.649 GHz.

As we mentioned earlier, it is imperative to ensure that the pump power is minimised while achieving the targeted gain, as a higher pump current would induce higher resistive loss and the coupled mode equations cannot accurately predict the performance of the KITWPA at high pumping levels. Therefore, we first investigate the relation of the pump power with the parametric gain for different number of total unit cells i.e., the total length of the amplifier, as shown in Figure 11(a). One striking observation here is that the gain does not pick off before *I*_p_ *≈* 0.3 *I*_0_ for all cases with different length of KITWPA, cascading from 50 to 150 unit cells, and the slope of the gain curves becomes steeper with longer transmission length i.e., the more unit cells there is, the faster the amplifier can reach maximum gain at lower pump power. However, the onset where the signal tone starts to experience gain does not occur until higher pump current values compared to the shorter amplifier option. This behaviour is consistent with the phase mismatch relation to the gain as explained in Section 2, and illustrated in Figure 11(b). One noted that the total phase mismatch ∆ _*ϕ*_ at low *I*_p_ for longer transmission length is further away from the zero axis compared to the shorter option, hence the onset at higher pump amplitude, even though the rate of approaching ∆ _*ϕ*_ = 0 axis is faster for longer length. These plots are important as it shows that to achieve moderate gain of 20 dB, it may not be necessary to opt for longer transmission line. As shown in Figure 11(a), once the amplifier is 100 unit cells long, we can achieve 20 dB gain at around *I*_p_ *≈* 0.4 *I*_0_, similar to 125 and 150 unit cells options. Since shorter transmission length will reduce risk of physical damage and results in smaller foot print, we therefore concluded that the optimal design in this case would be a KITWPA with 100 repeated cells. 

One would expect that the parametric gain would increase exponentially with *I*_p_ at all values, but as shown in Figure 11(a) this is not the case. The maximum gain plateaus at around 55 dB. This is because the signal amplitude at this pump current level is now higher than the pump current itself, hence the energy starts to flow backward from the signal to the pump. This is shown clearly in Figure 11(c) that depicts the case of a 100 cells amplifier, showing how the pump starts to deplete near *I*_p_ *≈* 0.56 *I*_0_ and reach a minimum at *I*_p_ *≈* 0.67 *I*_0_ where the signal amplitude peaks. After this point, the signal amplitude starts to deplete and the pump slowly regains its amplitude, hence the oscillating behaviour between the two tones as shown in Figure 11(a). One should note that the position of this peak gain is dependent on the total phase mismatch of the amplifier ∆ _*ϕ*_ as well, as illustrated in Figure 11(b), because this parameter is dependent on the pump amplitude as well. The maximum peak gain near 55 dB and its location are also dictated by the initial signal amplitude used in the calculation where in our case we assumed *I*_s_(0) = 10 ^–3^ *I*_p_, and with lower *I*_s_(0) the higher the maximum gain would be, and peak at higher *I*_p_, as the signal amplitude requires higher gain to be comparable to the pump amplitude.

**Figure 12.**
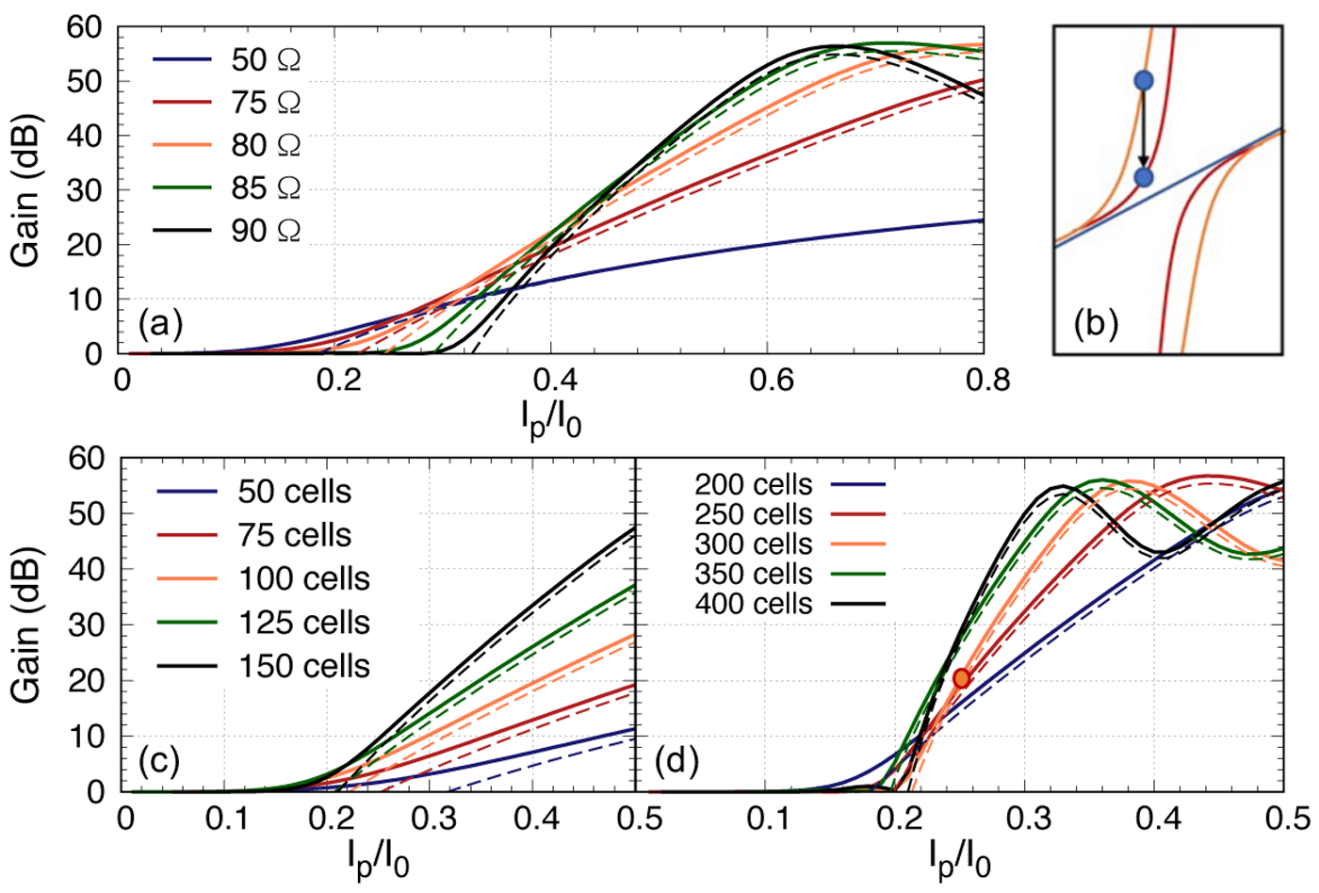
( **a**) The parametric gain plotted against pump power for a 100 unit cells KITWPA (Kinetic Inductance Travelling Wave Parametric Amplifier) with different *Z*_0_ value for the third loading, where I _p_ and I _0_ is the pump current and the critical current respectively. The gain onset starts earlier with lower pump power if the third loading *Z*_0_ is closer to the primary loading impedance. ( **b**) Sketch illustrates how the dispersion relation is differ if *Z*_03_ is decrease from 80Ω (orange) to 75Ω (red), therefore reduces the extra dispersion if the pump frequency remains at the same position. ( **c**) & ( **d**) Gain versus pump power for different length of KITWPA with *Z*_03_ = 75Ω. All curves were plotted with signal frequency of 9 GHz and pump frequency of 7.649 GHz.

One important observation of Figure 11(b) is that at the point where the peak gain occurs, the phase mismatch ∆ _*ϕ*_ ≠ 0. This implies that we may have over compensated the phase correction before the pump starts to deplete as the excursion of the dispersion relation is related to the ratio of the impedance of the third loading section to the primary loading section. Furthermore, we would like to explore if there is a method to further reduce the required pump current. As the dispersion diversion from the otherwise linear dispersion relation is directly related to the ratio of the third loading impedance with the primary loading section, another way to reduce the over-compensation of the dispersion is by reducing this impedance difference. Here we investigate if we can achieve higher gain with lower pump current by using different *Z*_0_ for the third loading. The result is shown in Figure 12, and we include the case of a linear amplifier without any periodic loading, notated as 50Ω curve in the plot. Again, we see that to achieve 20 dB gain, all the plots converge at *I*_p_ = 0.4 *I*_0_. However, from Figure 11 we learn that with longer line, the rate to achieve maximum gain is steeper, hence we can in fact increase the number of unit cells to achieve 20 dB gain with lower *I*_p_, and this is indeed the case (see Figure 12(b) and (c)), but at the expense that we now need close to 250 unit cells to reduce the *I*_p_ from 0.4 to 0.25. In short, it is possible to reduce the gain onset point to lower *I*_p_ value and hence reduce the required *I*_p_ value to achieve 20 dB gain but one would need much longer transmission length.

**Figure 13.**
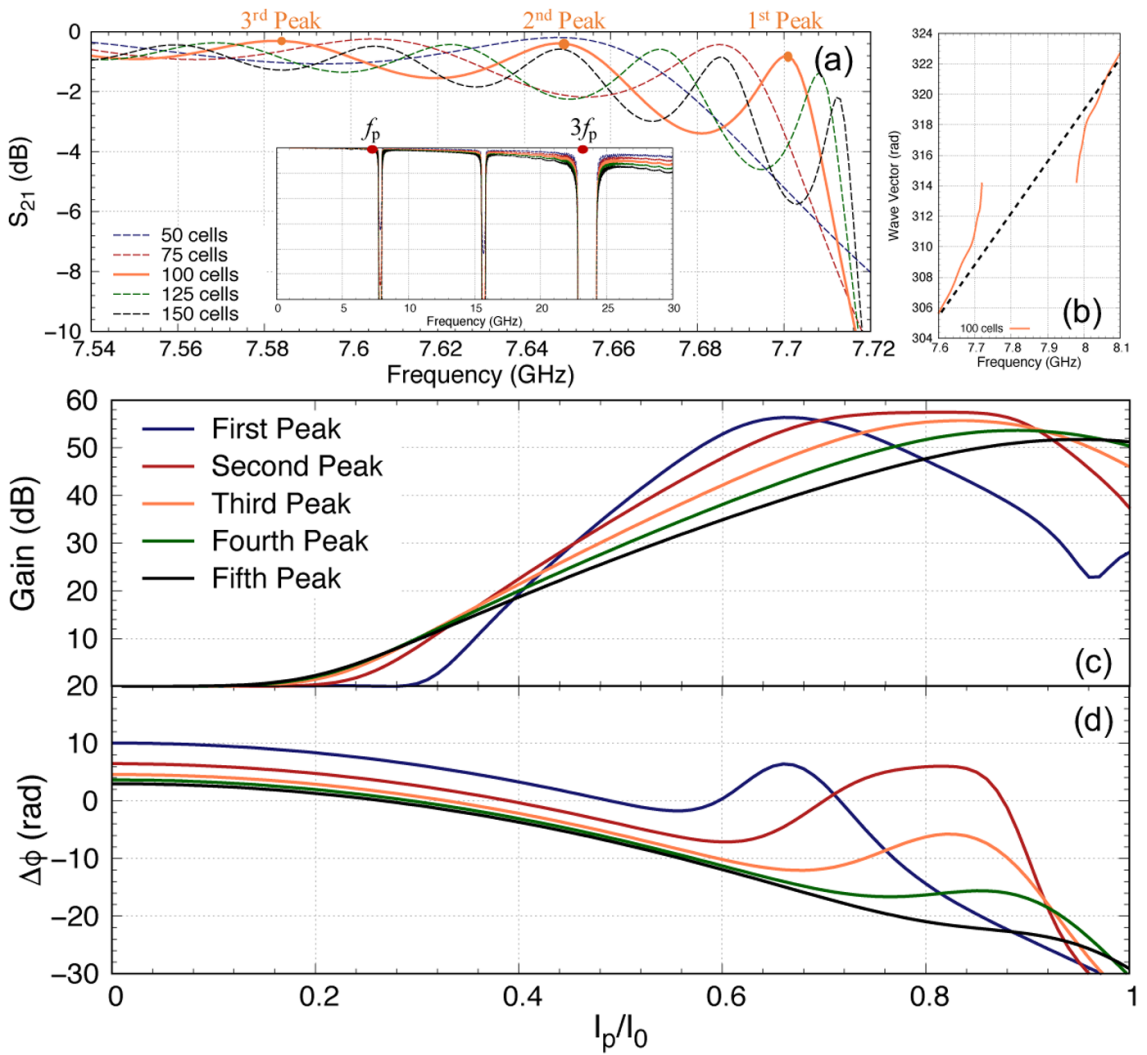
( **a**) The insertion loss of the KITWPAs (Kinetic Inductance Travelling Wave Parametric Amplifiers) with different lengths near the first stopband around 8 GHz, showing the peaks with lowest loss, where I _p_ and I _0_ is the pump current and the critical current respectively and *f*_p_ is the pump frequency ( **b**) The dispersion relation of the 100 unit cells KITWPA. ( **c**) The parametric gain and ( **d**) total phase mismatch of the 100 unit cells KITWPA with the pump frequency fixed at different peak positions. All curves were plotted with signal frequency of 9 GHz.

Another way we can reduce the overcompensation of the phase mismatch is by placing the pump frequency further away from the 8 GHz stopband to reduce the overcorrection and we explore this solution in Figure 13 for the case of 100 unit cells amplifier. The downside of this option is that it may result in a larger zero-gain gap. This differs from previous analysis where we always place the pump at the first peak closest to the stopband as shown in Figure 13(a) because the dispersion excursion is maximised at this point with the lowest insertion loss, here we explore the difference in gain behaviour if we place the pump frequency at subsequent peaks further away from the primary stopband. Note that this is another advantage of using the methodology described in this paper, where we can effectively capture the behaviour of the cascaded cells, which produce the ‘ringing’ effect near the stopbands, that is not possible with translation symmetry assumption used in most models reported in literature. So here, we investigate the same *I*_p_ vs gain relation by placing the pump at different frequencies to try to achieve ∆ _*ϕ*_ = 0 before the pump depletion kicks in. Note that the insert in Figure 13 shows exactly how we suppressed the pump’s third harmonics by having a large stopband at 3 *f*_p_.

**Figure 14.**
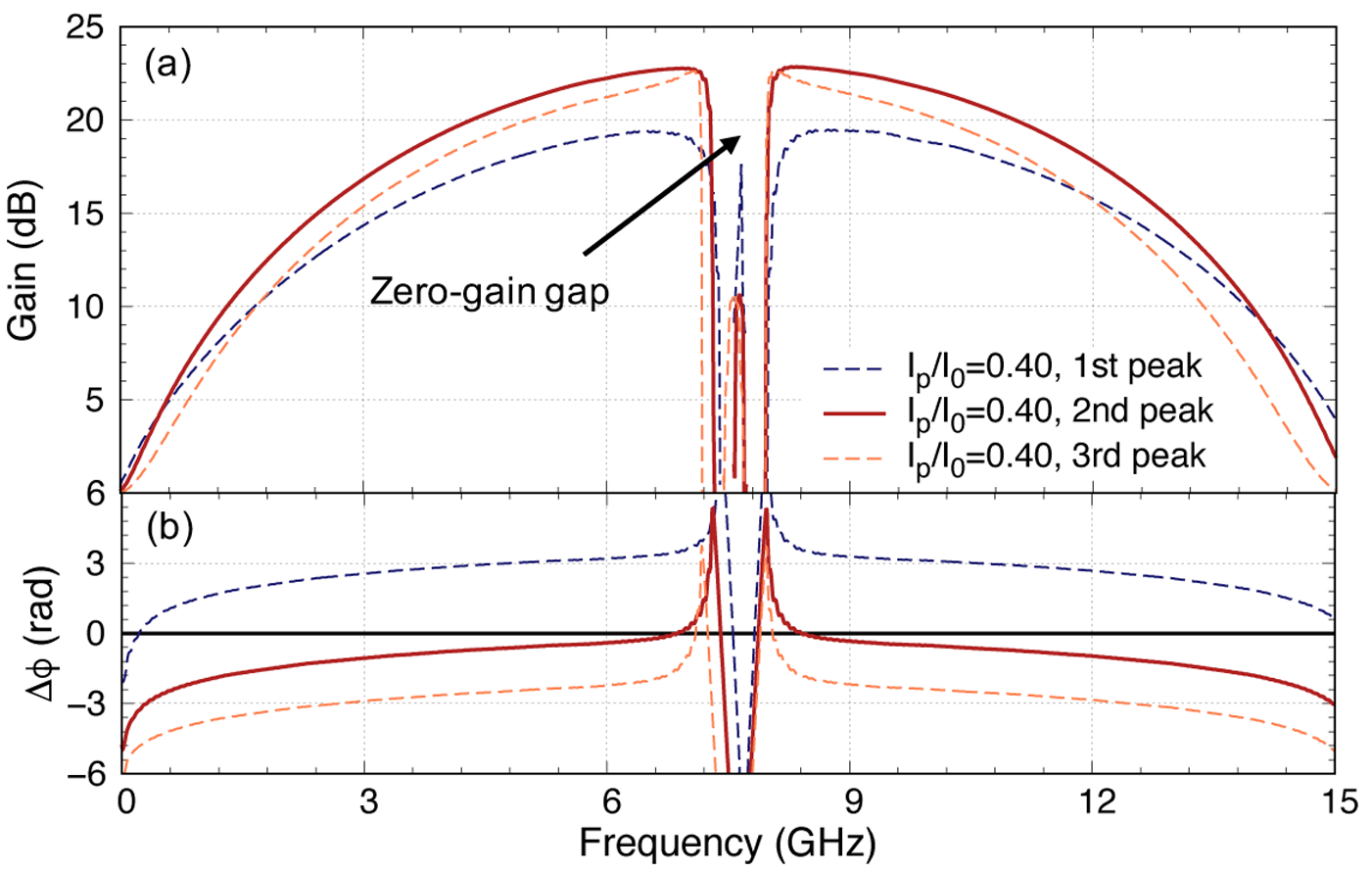
( **a**) The gain-bandwidth profile of a 100 unit cells KITWPA (Kinetic Inductance Travelling Wave Parametric Amplifier) with pump frequency placed at first, second and third peak of the insertion loss curve, where I _p_ and I _0_ is the pump current and the critical current respectively and ∆ _*ϕ*_ is the total phase mismatch. ( **b**) the total phase mismatch corresponding to ( **a**). The gain bandwidth is broadest with pump frequency placed at the second peak, where the total phase mismatch is closest to zero over the broadest bandwidth.

As we can observe from Figure 13(b) that by choosing different pump frequency, indeed we can achieve the onset point at lower *I*_p_ value, but to achieve 20 dB gain, we only reduce *I*_p_ by about 5%. It is clear therefore, the optimal case here if we pump the amplifier at the second peak where one note that ∆ _*ϕ*_ = 0 near *I*_p_ = 0.4 *I*_0_ now. The gain at this point starts to drop if we shift further away with higher peak number, as the total phase mismatch once again deviates away from the zero axis. The upside of this is that the second peak is only 0.05 GHz away from the first peak, hence would not widen the zero-gain gap too drastically. This combination is therefore the optimal design we can achieve with the CPW amplifier. In Figure 14, we plot the gain profile of this optimal KITWPA with 100 unit cells pumped at *I*_p_ = 0.4 *I*_0_ with the pump frequency located at the second peak, where ∆ _*ϕ*_ is closest to zero across the broadest bandwidth, hence achieving 20 dB gain with the broadest bandwidth as well.

### 5.4 Inverted microstrip with 50nm TiN film

Similar to Subsection 5.2, in this section we focus on the design of a compact KITWPA comprising 50nm thin TiN film in the form of inverted microstrip deposited on a sapphire substrate. Here, we further compare the use of different types of dielectric layer for inverted microstrip structure with silicon dioxide (SiO _2_) and amorphous silicon (aSi), apart from the use of silicon monoxide (SiO) shown earlier. The properties of these dielectric material, as well as their dimension required to achieve 50Ω characteristic impedance and the transmission loss is tabulated in Table 5. Note that for the case of aSi, with the original thickness of 100nm dielectric layer, we would need a strip width of 1.3 µm to achieve 50Ω ( *L*_*λ*/2_ =255 µm). Therefore we increase the dielectric thickness ( *t*_diel_) to 200nm such that the width is now closer to 2 µm, to ease the fabrication.

**Table 5.** Electromagnetic characteristics of 50nm thick TiN (Titanium Nitride) inverted microstrip on sapphire with different dielectric layers, where _r_ is the relative dielectric constant, tan *δ* is the loss tangent, *ρ* is the resistivity, *t*_diel_ is the thickness of the dielectric, Ref. is the references, *L*_*λ*/2_ is the half wavelength length, S _21_ is the insertion loss, SiO is silicon monoxide, SiO2 is silicon dioxide, and aSi is amorphous silicon. The total length used here to calculate the S _21_ is 70 wavelengths at 24 GHz.

	Inverted microstrip		
	SiO	SiO _2_	aSi
_r_	5.8	4.0	11.9
tan *δ*	0.004	3.0×10 ^−4^	2.2×10 ^−5^
*ρ*	∞	∞	15 kΩcm
*t*_diel_	100nm	100nm	200nm
Ref.	52	53	
Width	1.95 μm	2.40 μm	1.88 μm
*L*_*λ*/2_	336 μm	400 μm	340 μm
S _21_:10 GHz	–3.75 dB	–1.08 dB	–1.61 dB
S _21_:30 GHz	–16.72 dB	–8.58 dB	–8.80 dB

Comparing the three different dielectric materials, one immediately notes that the use of SiO incurs much higher loss than SiO _2_ and aSi, due to the high loss tangent of SiO. In principle, aSi should possess less transmission loss compared to SiO _2_ as the loss tangent is an order of magnitude lower, but due to the moderately high resistivity, the loss now become comparable to SiO _2_. Since the strip width and length is largely similar for all three types of dielectric, we therefore opt for SiO _2_ as it has the lowest loss property. Furthermore, fabrication of aSi requires special development procedure and it’s not commonly used in the field of microwave cryogenic engineering. It is worthwhile noting that although the effective length of SiO _2_ line is slightly longer, it is still shorter compared to the CPW line by close to a factor of three. Without the need for capacitive stubs, we can therefore produce a much more compact amplifier design using this transmission line configuration.

Similar to the CPW KITWPA design, we engineer the dispersion relation of the inverted microstrip KITWPA through the use of periodic loading scheme, where the dimensions and characteristic impedances of each loading sections and the primary 50Ω section are shown in Table 6. Here, we include the analysis for SiO as well as SiO _2_ to provide comparison and information with regards to how the loss mechanism may affect the gain-bandwidth product of the KITWPA.

**Table 6.** Dimension of the transmission line forming a single unit cell for the inverted microstrip TWPA (Travelling Wave Parametric Amplifier) on a sapphire substrate with different dielectric layers, where *Z*_0_ is the characteristic impedance, SiO is silicon monoxide and SiO _2_ is silicon dioxide.

	SiO		
	Primary Line	1 ^st^ Loading	3 ^rd^ Loading
*Z*_0_	50Ω	40Ω	25Ω
Width	1.95 μm	2.60 μm	4.60 μm
Length	336 μm	33.6 μm	33.6 μm
	SiO _2_		
	Primary Line	1 ^st^ Loading	3 ^rd^ Loading
*Z*_0_	50Ω	40Ω	25Ω
Width	2.40 μm	3.20 μm	5.40 μm
Length	400 μm	40 μm	40 μm

**Figure 15.**
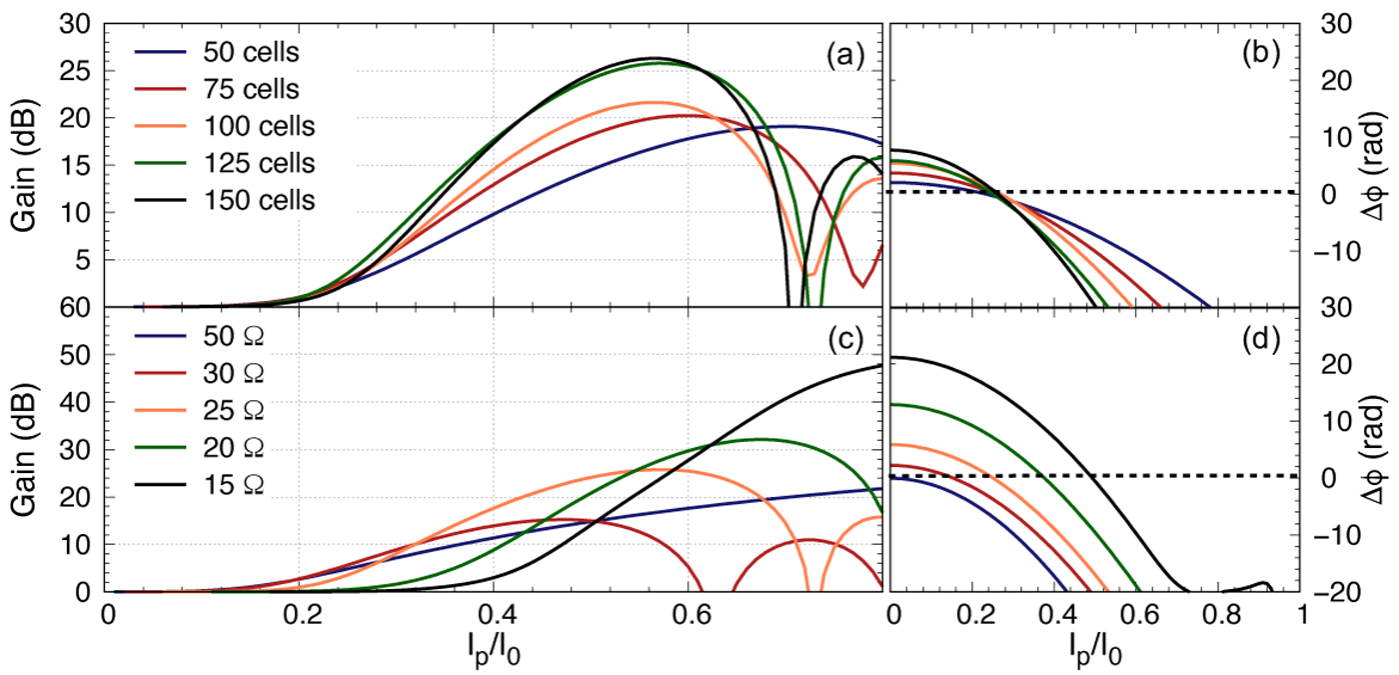
( **a**) The relation of parametric gain and ( **b**) phase mismatch plotted against the pump power for different length of the inverted microstrip (Inv. M.S.) KITWPA (Kinetic Inductance Travelling Wave Parametric Amplifier) with silicon monoxide dielectric layer,) with the third loading impedance of 25Ω. ( **c**) The parametric gain and ( **d**) phase mismatch plotted against pump power for a 125 unit cells KITWPA with different *Z*_0_ value for the third loading. All curves were plotted with signal frequency of 9 GHz and pump frequency of 7.83 GHz.

We first investigate the relation between the pump power and the gain of the inverted microstrip KITWPAs with SiO dielectric layer, as shown in Figure 15. One notes immediately that the maximum gain can now only achieve close to 25 dB instead of *∼*55 dB for the CPW KITWPA with 100nm TiN film, because of the high loss of the inverted microstrip line e.g., with 100 unit cells, we are just about to reach 23 dB gain maximally. Increasing the number of unit cells improves the situation slightly but does not dramatically alter the gain profile. For example, the gain curve for both cases with 125 and 150 unit cells are largely similar, because as the transmission length gets longer, the resistive loss starts to dominate over the parametric gain, hence starts to lower the total gain of the amplifier after 150 unit cells as the pump amplitude is now weaken by the additional loss. From Figure 15(a) and (b), we observed that to achieve 20 dB gain, we would need higher pump power as well, between 40–60% of the *I*_0_ for different number of unit cells. Furthermore, the phase mismatch curves for all 5 different lengths cross the zero-axis near *I*_p_ = 0.25 *I*_0_, and diverge from the zero-axis further with longer amplifier length, indicating that we are now under-compensating the phase correction. Both this and the additional loss of the inverted microstrip line contribute to the low gain obtainable by the amplifier despite the use of higher pump power. Therefore, it is important to consider the loss behaviour of the transmission line utilised, because with high loss there may exist an optimal parametric length required, where a shorter line results in low parametric gain, while a longer line incurs high loss which again lowers the overall gain. In the case presented here, it is obvious that we can achieve the best result (highest gain with lowest *I*_p_ and shortest length) with 125 unit cells.

As described earlier, we can alter the impedance of the third loading to increase the dispersion divergent to accumulate more phase correction. This approach is shown in Figure 15(c) and (d), where indeed with lower *Z*_0_ (hence larger difference compared to the characteristic impedance of the primary 50Ω line) the ∆ _*ϕ*_ curves can now reach the zero axis at higher pump level, hence the improvement in the peak gain as well since the parametric gain is improved now. However, the onset of gain now shifted to higher pump amplitude position, hence higher pump current would be needed to achieve the same gain for lower loading impedance, which could be detrimental as explained earlier. Therefore, we conclude that in this particular case, a 125 unit cells KITWPA with third loading impedance of 25Ω is the optimal solution here. This again stressing that in the case of high loss, there indeed exists an optimal solution, where the designer should aim to achieve the desired peak gain that coincides with the maximum gain achievable. In this scenario, the amplitude of the pump is optimum in overcoming the resistive loss and provide high enough power for exciting sufficient parametric gain.

**Figure 16.**
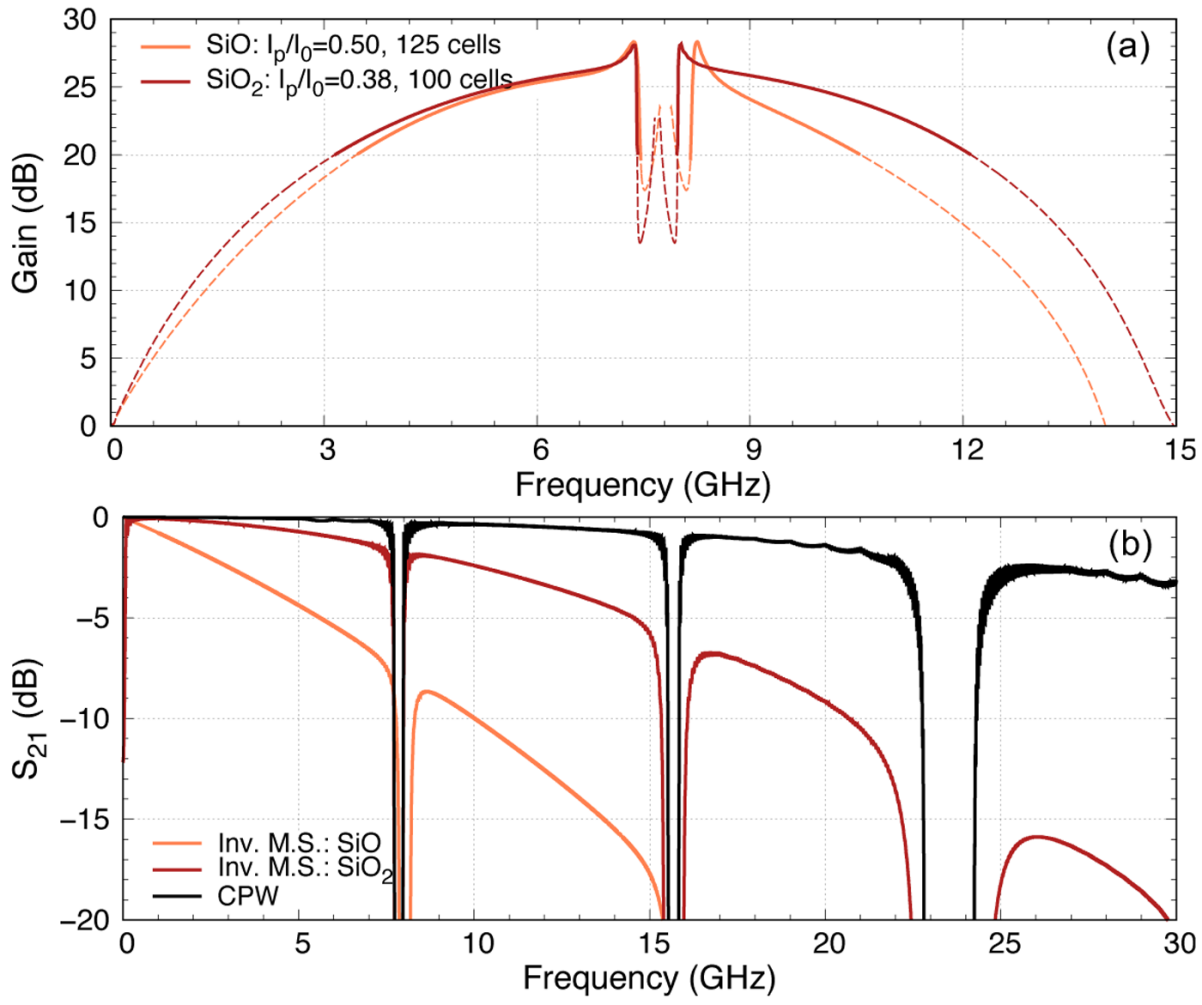
( **a**) Gain profile for the optimised inverted (inv.) microstrip KITWPA (Kinetic Inductance Travelling Wave Parametric Amplifier) with SiO (silicon monoxide) and SiO _2_ (silicon dioxide) dielectric layer. ( **b**) The S _21_ transmission profile of both inverted microstrip designs compared to the CPW (coplanar waveguide) design presented earlier.

Applying the same analysis and following the similar steps described in Subsection 5.2 including optimising the pump frequency, we shows the predicted gain profiles for both SiO and SiO _2_ designs in Figure 16. Both designs utilise a third loading impedance of 25Ω with amplifier length of 125 unit cells for the SiO design and 100 unit cells for the SiO _2_ design respectively. The pump current required to achieve the shown gain profile are *I*_p_/ *I*_0_=0.50 and 0.38 for SiO and SiO _2_ design. As expected, due to the lower loss of the SiO _2_ dielectric layer, we can now achieve the similar peak gain with fewer unit cells than the CPW design and lower pump current close to the one utilised for the CPW KITWPA. An interesting feature of these gain profile is that the gain is asymmetric for the SiO design, due to the extremely high loss at the high frequency end, as shown in Figure 16(b). This effectively results in a narrower bandwidth where we can only achieve higher than 20 dB gain from 3.5–10.5 GHz for the SiO design, whilst broader from 3–12 GHz for the SiO _2_ amplifier. This exercise once again shows the important of including the loss in the simulation of the KITWPA, as it inevitably has a large effect on the performance of the KITWPA. One important note with regards to the high loss designs is that as the resistive loss is frequency dependent, therefore it would be difficult to operate the KITWPA at higher frequencies, including the option of pumping the amplifier at the second stopband, or operating with the DC-biased 3WM mode, as the pump amplitude would be severely attenuated because the pump is now placed at a higher frequency position. This would mean that most of the pump power is converted into resistive heat loss instead of transferring to the signal wave for amplification.

**Figure 17.**
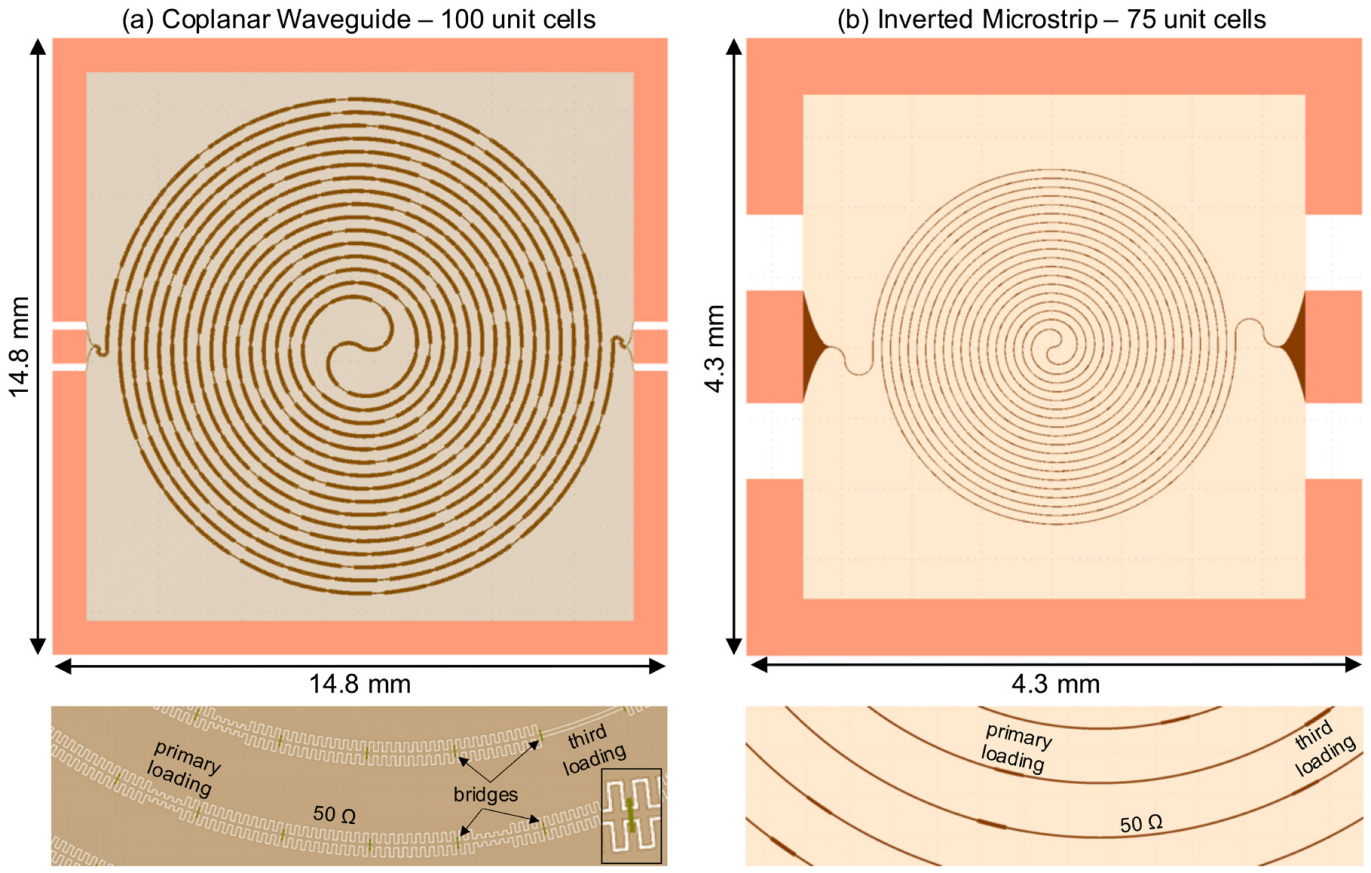
( **a**) The layout of the optimised CPW (coplanar waveguide) KITWPA (Kinetic Inductance Travelling Wave Parametric Amplifier) with 100 repeated unit cells wrapped in a double spiral fashion. The zoomed-in image below shows the various sections making up the unit cells, and a further zoom-in image showing the bridge. ( **b**) Similar layout for the optimised inverted microstrip KITWPA.

In Figure 17, we show the actual layout of the two optimised KITWPA designs described in this paper, which have very similar gain-bandwidth product: (a) the low loss CPW KITWPA with 100nm TiN film on sapphire substrate, and (b) the compact inverted microstrip KITWPA with 50nm TiN film with SiO _2_ dielectric layer. As mentioned earlier, our simulation for the CPW TWPA includes the existence of the equipotential bridges as shown in the enlarged image of Figure 17, which also illustrate how the primary 50Ω section, the first and third loading section are populated across the entire amplifier. In these particular designs, we opt for winding the long transmission line into a double-spiral form for compactness and low cross talk between the adjacent transmission line. Comparing the two, one immediately notes that the inverted microstrip design is much more compact with a foot print of 3.5×3.5mm compared to the CPW amplifier, which is about 18× smaller in size, dominated mainly by the size of the bonding pads required near the two ends of the amplifier.

**Figure 18.**
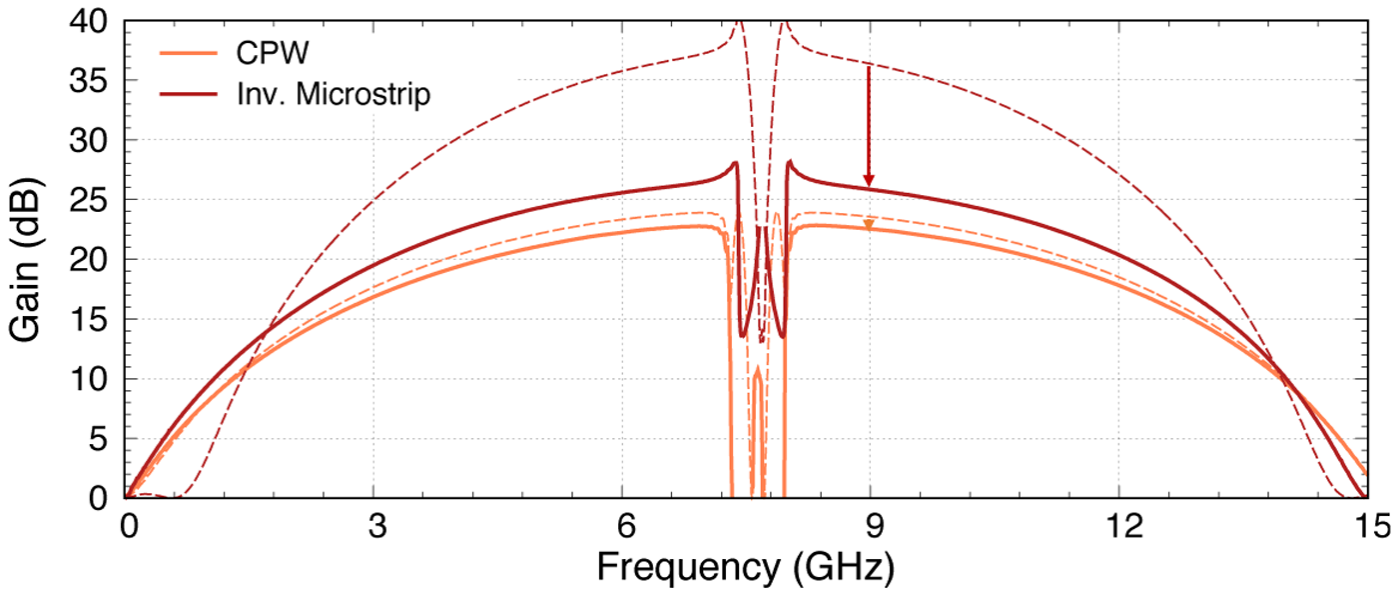
Comparison of gain profile for both CPW (coplanar waveguide) and inverted microstrip KITWPAs (Kinetic Inductance Travelling Wave Parametric Amplifiers) with (solid lines) and without (dashed lines) taking into account the loss in the form of *α*_p,s,i_. Note that the dashed lines are effectively the actual parametric gains of the device, and with loss the gain would be reduced as expected.

Finally, we should emphasise that the reason both KITWPAs design can obtain similar high gain despite the difference in transmission loss is because the higher loss inverted microstrip KITWPA utilises a higher kinetic inductance 50nm TiN film, hence would have higher parametric gain to compensate the additional loss. We demonstrate the effect of properly taking into account the transmission line loss in Figure 18, where we can see that for the lower loss CPW KITWPA, the predicted gain profile is largely similar with or without loss; but for the inverted microstrip KITWPA, if the loss is not included in the calculation, we would over estimate the gain by close to 12 dB. This again shows the important of capturing all the information with regards to the transmission line so that we can accurately predict the performance of the KITWPAs.

Although it may appear that the inverted microstrip design is a better option in this case since it is much compact and less susceptible to physical damage, we should point out that if we take into consideration that with high resistive loss and pump current, the devices may generate more resistive heat as well as additional noise in the form of Johnson noise, then the inverted microstrip design may have inferior noise performance compared to the CPW design. Since most of the energy of the system is carried by the pump wave when the KITWPA is in operation, we can estimate the noise power via $I_{\text{rms}}^{2}R$ , where the resistance of the wire is given by ℜ( *γ*_p_ *Z*_0_). As our TiN film have a critical current of *∼*3 mA, and with the corresponding value of *I*_p_/ *I*_0_ for each cases, we would arrive at 10.6 µW for inverted microstrip design compared to 1.3 µW for the CPW amplifier, which is almost 10 *×* higher. Hence, there is inevitably a compromise between a small footprint KITWPA and low loss/noise design, and depending on the application, the designer may opt for one over the other based on the preferences.

Nevertheless, our designed KITWPAs are expected to achieve the targeted 20 dB gain, in fact higher gain and hence broader bandwidth with fewer unit cells compared to the results reported in most literatures, demonstrating the feasibility of our method to optimise the performance of a KITWPA. For example, comparing to the design of the KITWPA presented in 22 which only managed to achieve 9 dB gain with 140 unit cells, we manage to reach a peak gain near 25 dB with shorter transmission length around 100 to 125 unit cells. Note that this is close to 30 *×* improvement in amplification of the signal strength with a slightly compact design. Comparing to 7 which operates at the second stopband, again the said amplifier would need 224 unit cells with a pump current much higher than 0.4 *I*_0_ to reach peak gain of 10 dB. Therefore, we trust that we have illustrates that there is indeed room for improvement with the existing design of KITWPA in the literature, and with our approach we could maximise the gain with shortest transmission length and lowest required pump current.

## 6 Conclusions

In this paper, we have described in detail our procedures and analyses carried out to identify the most suitable superconducting and non-conducting materials required to form a KITWPA, as well as the superconducting transmission line topologies and the operational parameter spaces, to produce a high-gain low loss amplifier that can potentially achieve quantum limited noise performance across broad bandwidth with small foot print. In the first part of the paper, we presented a novel method that allows us to fully capture the electromagnetic behaviour of all components making up the amplifier such as the effect of superconductivity, loss tangent, fringing fields etc, and therefore better predict the performance of the KITWPA. This methodology is also very flexible, taking advantages of the versatility of commercial electromagnetic and microwave circuit softwares, and the fast computing coupled-mode equation models, which allows us to explore effect of many different type of materials and transmission line structures that are difficult to simulate using traditional method.

In the second part of the paper, we presented our extensive studies of comparing the performance of KITWPA comprising different type of superconducting materials, in search of a high kinetic inductance and low surface resistance superconducting thin film. We explored their advantages and disadvantages in terms of dependent on operational frequency range, normal resistivity, critical temperature etc, including an engineered TiN film. We found that as long as the thin film behaves in the BCS regime, the choice of the superconducting material has only marginal effect on the performance of the KITWPA. The required kinetic inductance and low surface resistance of any film can in fact be obtained by choosing the appropriate film thickness, as long as it is feasible to fabricate using modern photo-lithography and/or e-beam techniques.

Next, we investigate the most suitable transmission line topology to provide the lowest insertion loss, shortest transmission length and the ease of fabrication (sensible circuit dimensions) to form a TiN KITWPA. We have realised that there is a compromise between achieving low transmission loss with the compactness of the device. A CPW amplifier with thicker film can indeed achieve low insertion loss, but in order to minimise cross talk and the need for capacitive stubs to reach 50Ω characteristic impedance inevitably hinders the compact packing of the transmission line. An inverted microstrip with thinner film, on the other hand, can produce a much shorter effective transmission length than the CPW option, but the higher resistive loss could potentially result in unwanted higher noise performance.

Finally, we report on the design of the two KITWPAs described above, in terms of optimal length required to achieve higher than 20 dB gain with minimal pump power, including the proper placement of the pump frequency and optimising the configuration of the periodic loading scheme to reach exponential gain. In that section, we further illustrated that once the design is optimised, it is possible to operate the KITWPA in different modes, such as operating at higher frequency range, cover broader bandwidth with DC-biasing the device and the potential use of the amplifier in the non-linear regime for high dynamic range applications. We conclude the paper by presenting and comparing the performance of the two optimal KITWPAs including their actual layout and their gain-bandwidth product respectively, for the CPW and the inverted microstrip amplifier, which we pay special attention to the effect of transmission loss on the gain profile of our parametric amplifiers. For both designs, we successfully achieved a predicted gain that is higher than 20 dB with close to 100% bandwidth product with small device area and lower pump current, compared to the existing designs reported in the literature, hence verify the feasibility of our optimised approach for designing a KITWPA.

## Data Availability

Figshare: Engineering the Thin Film Characteristics for Optimal Performance of Superconducting Kinetic Inductance Amplifiers using a Rigorous Modelling Technique. https://doi.org/10.6084/m9.figshare.c.6034133.v1 ^51^. This project contains the following underlying data, organised by ‘dataset’ within the Figshare collection: Dataset for Figure 2 (Raw datasets in .dat for each panel in Figure 2) Dataset for Figure 3 (Raw datasets in .txt for each panel in Figure 3) Dataset for Figure 4 (Raw datasets in .txt for each panel in Figure 4) Dataset for Figure 5 (Raw datasets in .txt for each panel in Figure 5) Dataset for Figure 6 (Raw datasets in .txt for each panel in Figure 6) Dataset for Figure 7 (Raw datasets in .txt for each panel in Figure 7) Dataset for Figure 8 (Raw datasets in .txt for each panel in Figure 8) Dataset for Figure 10 (Raw datasets in .dat for each panel in Figure 10) Dataset for Figure 11 (Raw datasets in .dat for each panel in Figure 11) Dataset for Figure 12 (Raw datasets in .dat for each panel in Figure 12) Dataset for Figure 13 (Raw datasets in .dat and .csv for each panel in Figure 13) Dataset for Figure 14 (Raw datasets in .dat for each panel in Figure 14) Dataset for Figure 15 (Raw datasets in .dat for each panel in Figure 15) Dataset for Figure 16 (Raw datasets in .dat for each panel in Figure 16) Dataset for Figure 17 (Raw datasets in .txt and .csv for each panel in Figure 17) Dataset for Figure 19 (Raw datasets in .dat and .txt for each panel in Figure 19) Figshare: Engineering the Thin Film Characteristics for Optimal Performance of Superconducting Kinetic Inductance Amplifiers using a Rigorous Modelling Technique. https://doi.org/10.6084/m9.figshare.c.6034133. v1 ^51^. This project contains the following extended data, organised by ‘dataset’ within the Figshare collection: Plotting script for Figure 2 (Gnuplot script in .gnu that reads & processes the raw data and plots each panel in Figure 2) Plotting scripts for Figure 3 (Gnuplot scripts in .gnu that read & process the raw data and plot each panel in Figure 3) Plotting script for Figure 4 (Gnuplot script in .gnu that reads & processes the raw data and plots each panel in Figure 4) Plotting scripts for Figure 5 (Gnuplot scripts in .gnu that read & process the raw data and plot each panel in Figure 5) Plotting scripts for Figure 6 (Gnuplot scripts in .gnu that read & process the raw data and plot each panel in Figure 6) Plotting script for Figure 7 (Gnuplot script in .gnu that reads & processes the raw data and plots each panel in Figure 7) Plotting scripts for Figure 8 (Gnuplot scripts in .gnu that read & process the raw data and plot each panel in Figure 8) Plotting script for Figure 10 (Gnuplot script in .gnu that reads & processes the raw data and plots each panel in Figure 10) Plotting scripts for Figure 11 (Gnuplot scripts in .gnu that read & process the raw data and plot each panel in Figure 11) Plotting scripts for Figure 12 (Gnuplot scripts in .gnu that read & process the raw data and plot each panel in Figure 12) Plotting scripts for Figure 13 (Gnuplot scripts in .gnu that read & process the raw data and plot each panel in Figure 13) Plotting scripts for Figure 14 (Gnuplot scripts in .gnu that read & process the raw data and plot each panel in Figure 14) Plotting scripts for Figure 15 (Gnuplot scripts in .gnu that read & process the raw data and plot each panel in Figure 15) Plotting scripts for Figure 16 (Gnuplot scripts in .gnu that read & process the raw data and plot each panel in Figure 16) Plotting script for Figure 17 (Gnuplot script in .gnu that reads & processes the raw data and plots each panel in Figure 17) Plotting script for Figure 19 (Gnuplot script in .gnu that reads & processes the raw data and plots each panel in Figure 19) Data are available under the terms of the Creative Commons Attribution 4.0 International license (CC-BY 4.0).

## Footnotes

^1^We exclude Al and Nb here because the extremely low *L*_s_ value gives rise to an extremely high ratio even with a relatively low *R*_s_ value. Also, altering the film thickness would simply shift the entire plot along the vertical axis with higher *L*_s_/ *R*_s_ ratio for thinner films, as indicated in Figure 3.

^2^The resistivity was measured at room temperature and corrected by a 1.1 coefficient (obtained from previous experiments) to find the resistivity at cryogenic temperature before the transition temperature.

^3^The 10 GHz curve is exactly the same curve as shown in Figure 7

^4^Even with 100nm TiN that posses lower surface resistance, the central strip to gap width aspect ratio is still formidably high, closed to 10:1 for sapphire and silicon, and 60:1 for quartz, which is not realistic and it may excite unwanted modes even with equipotential bridges. The scenario is even worse with thinner film that have higher inductance.

## Software availability

A third-party proprietary software, Ansys ^®^Electronics Desktop 2018.1.0 (Release 19.1.0), was used to obtain the electromagnetic properties of the film under study, such as the wavelength, insertion loss, wave vector and characteristic impedance tabulated in the manuscript and the dataset listed in the *Data availability* statement. Due to the complexity of Ansys ^®^Electronics Desktop, there are no freely available open source alternatives to replace the full functionality of this *em* software and that can perform the same simulation (to our knowledge). Nevertheless, we have described the primary methodology behind the software and have provided the associated output data and analysis code.
