# Supplementary material for: Engineering the thin film characteristics for optimal performance of superconducting kinetic inductance amplifiers using a rigorous modelling technique
Source: Open Res Eur. 2023 Sep 13;2:88. Originally published 2022 Jul 19. [Version 2] doi: 10.12688/openreseurope.14860.2 (PMC10521635; doi:10.12688/openreseurope.14860.2)
Supplement: Peer review history

# Reviewer report: Reviewer response for version 2

**DOI:** 10.21956/openreseurope.17916.r35100

**Published:** 2023-10-12

**Authors:** Fausto Patricio Mena

**Affiliations:** 1 Electrical Engineering Department, University of Chile, Santiago, Chile; 2 National Radio Astronomy Observatory, Charlottesville, VA, USA

The authors have addressed all my comments. No further revision is needed.

Is the study design appropriate and does the work have academic merit?

Yes

Is the work clearly and accurately presented and does it cite the current literature?

Partly

If applicable, is the statistical analysis and its interpretation appropriate?

Not applicable

Are all the source data underlying the results available to ensure full reproducibility?

Yes

Are the conclusions drawn adequately supported by the results?

Partly

Are sufficient details of methods and analysis provided to allow replication by others?

Yes

Reviewer Expertise:

NA

I confirm that I have read this submission and believe that I have an appropriate level of expertise to confirm that it is of an acceptable scientific standard.

# Reviewer report: Reviewer response for version 2

**DOI:** 10.21956/openreseurope.17916.r35102

**Published:** 2023-10-02

**Authors:** Wenlei Shan

**Affiliations:** 1 Advanced Technology Center (ATC), National Astronomical Observatory of Japan (NAOJ), Tokyo, Japan

The authors have responded to all the comments that I made and made necessary modifications in the revision. I suggest approval of this version.

Is the study design appropriate and does the work have academic merit?

Yes

Is the work clearly and accurately presented and does it cite the current literature?

Partly

If applicable, is the statistical analysis and its interpretation appropriate?

Not applicable

Are all the source data underlying the results available to ensure full reproducibility?

Partly

Are the conclusions drawn adequately supported by the results?

Partly

Are sufficient details of methods and analysis provided to allow replication by others?

Partly

Reviewer Expertise:

Superconducting electronics, Radio Astronomical Techniques

I confirm that I have read this submission and believe that I have an appropriate level of expertise to confirm that it is of an acceptable scientific standard.

# Reviewer report: Reviewer response for version 2

**DOI:** 10.21956/openreseurope.17916.r35101

**Published:** 2023-09-22

**Authors:** Martina Esposito

**Affiliations:** 1 CNR, SPIN Institute, Naples, Italy

The authors satisfactory addressed all my previous comments and I have no further comments to make.

Is the study design appropriate and does the work have academic merit?

Yes

Is the work clearly and accurately presented and does it cite the current literature?

Partly

If applicable, is the statistical analysis and its interpretation appropriate?

Not applicable

Are all the source data underlying the results available to ensure full reproducibility?

Yes

Are the conclusions drawn adequately supported by the results?

Yes

Are sufficient details of methods and analysis provided to allow replication by others?

Yes

Reviewer Expertise:

superconducting quantum devices, traveling parametric amplifiers

I confirm that I have read this submission and believe that I have an appropriate level of expertise to confirm that it is of an acceptable scientific standard.

# Reviewer report: Reviewer response for version 1

**DOI:** 10.21956/openreseurope.16055.r30185

**Published:** 2022-11-15

**Authors:** Martina Esposito

**Affiliations:** 1 CNR, SPIN Institute, Naples, Italy

The manuscript presents an interesting modelling method for KTWPA devices capturing the effect of both the adopted materials and the chosen topology by combining the use of 3D em-simulation software and the solution of coupled differential equations for the description of modes propagation.

 First, the modelling methodology is presented. Subsequently, the authors focus on modelling different thin film materials and different transmission line topologies. Finally, two possible designs are discussed in details and the results of model’s predictions are compared with experimental results present in the literature.

 The presented method is certainly interesting, and this work provides useful guidelines for designing KTWPAs.

 However, in the present form, it results a bit difficult for the reader to catch the key results/achievements. Sections 3, 4 and 5 are very long with many figures, but probably some repetitions are present, for example different figures stressing similar aspects. I think the manuscript could strongly benefit from a careful revision with the aim to emphasising more the main results and summarizing in a more concise way the secondary aspects.

 Specific comments are given below:

**Abstract and Summary**

 I don’t think it appropriate to define KTWPAs as a “new” variant of superconducting amplifiers, considering that the first device obtained with  modern nanofab methods was demonstrated in 2012: Nat. Phys. 8, 623–627 (2012).

**Summary**

 “As the amplifier is formed using the superconducting thin”. Please check language/grammar.

**Introduction**

 The list of references for experimentally demonstrated JTWPAs is not updated. Please check and possibly update the reference list according to state-of-the-art.

**Methods**

 “If a weak signal at frequency  is detected”. Confusing wording here: in order to get amplification, a weak signal should be 'applied' at input before being 'detected' at output.

 Page 4 (end of first column): please check the correct use of punctuation, here and for the entire manuscript.

 Page 5: it would be interesting if the authors could provide more details on the computation time typically required for the full modelling of the single unit cell.

 Page 5: in order to obtain the results reported in the figures of section 5, are equation (3) and (4) solved numerically? Or, an analytic solution, under certain conditions, is adopted? This is a very interesting aspect; however, it is not very clear in my opinion in the manuscript and should be clarified.

 Fig 1: one may deduce that a second loading section is also included in the design, however this does not appear in Fig 1 sketch (only first and third loading sections are mentioned), can the authors please clarify?

 Page 6: how is the contribution from the  estimated?

 Methods: could the modelling method explained in section 2 be adapted for JTWPAs? it would be interesting if the authors could comment about this possibility.

 Please check that when an acronym is used for the first time, the definition is also reported. Example (MIKIDs in page 7).

 Page 7: “From the plot, it clearly indicates that”. Please check language/grammar.

**Section 3**

 How are plots (continuous lines) in fig from 2 to 8 obtained? If analytical expressions are used, can the authors please explicitly report the adopted analytical expressions for *R _s_*, *L _s_* and *p _s_*? Reporting the analytical expressions could help in possibly removing some of the figures (especially the ones showing secondary aspects, that can be equivalently well described with a formula).

 Fig 6: The authors should add or at least discuss error bars for the experimental points.

 Page 10 “Therefore, it would be indicative to perform the same analysis at a fixed operational frequency instead of fractional frequency”. I totally agree with this. The authors could indeed focus directly on this kind of analysis (at fixed operational frequency) which is the useful one for the designing a device in a given frequency range. This will probably help in making the manuscript shorter and it will be easier for the reader to appreciate the key results.

 Not necessary to repeat the acronym definition, the authors can define it just the first time that the acronym is used (general comment for all the manuscript). Example (KITWPA).

 Page 16: “Therefore, we are confident that our proposed method can accurately predicts the gain-bandwidth performance of a KITWPA.” Very nice benchmark of the method. Does the same kind of validation check work for micro-strip geometries?

**Section 5.3**

 Is the adopted modelling method considering the frequency-dependence of dielectric losses? Can the authors please give more details of this aspect? This is particularly relevant for the micro-strip geometry.

**Section 5.3 **“if the signal-to-noise ratio of the incoming signal can be estimated a priori.” What does this exactly mean? How do the authors suggest doing that?

 Page 22 (last paragraph in first column):  This sentence is confusing, please check.

 Fig. 19: Very interesting comparison!

 Page 25: it would be useful if the authors could discuss this noise comparison also in terms of expected number of photons above the standard quantum limit for the TWPA added noise.

 Page 25: The authors should be careful when comparing theoretical prediction with experimental results. For example, instead of using the expression “successfully achieve” I believe it would be more appropriate to say "are predicted to achieve"/"are expected to achieve" or similar expressions. I have the same language comment for the subsequent comparison with ref 22 and 7, since in the current manuscript no experimental data are presented.

 Last paragraph of section 5: Please check language/grammar.

Is the study design appropriate and does the work have academic merit?

Yes

Is the work clearly and accurately presented and does it cite the current literature?

Partly

If applicable, is the statistical analysis and its interpretation appropriate?

Not applicable

Are all the source data underlying the results available to ensure full reproducibility?

Yes

Are the conclusions drawn adequately supported by the results?

Yes

Are sufficient details of methods and analysis provided to allow replication by others?

Yes

Reviewer Expertise:

superconducting quantum devices, traveling parametric amplifiers

I confirm that I have read this submission and believe that I have an appropriate level of expertise to confirm that it is of an acceptable scientific standard, however I have significant reservations, as outlined above.

# Response

**Published:** 2023-09-01

**Authors:** Boon Kok Tan

**Affiliations:** University of Oxford, UK

Dear Dr. Esposito,   Thank you for taking your time to review the article thoroughly. My sincere apology for the long delay in responding. The responses for all your very useful comments are as below:  

 The manuscript presents an interesting modelling method for KTWPA devices capturing the effect of both the adopted materials and the chosen topology by combining the use of 3D em-simulation software and the solution of coupled differential equations for the description of modes propagation.

 First, the modelling methodology is presented. Subsequently, the authors focus on modelling different thin film materials and different transmission line topologies. Finally, two possible designs are discussed in details and the results of model’s predictions are compared with experimental results present in the literature.

 The presented method is certainly interesting, and this work provides useful guidelines for designing KTWPAs.

 However, in the present form, it results a bit difficult for the reader to catch the key results/achievements. Sections 3, 4 and 5 are very long with many figures, but probably some repetitions are present, for example different figures stressing similar aspects. I think the manuscript could strongly benefit from a careful revision with the aim to emphasising more the main results and summarizing in a more concise way the secondary aspects.

 Specific comments are given below:

**Abstract and Summary**

 I don’t think it appropriate to define KTWPAs as a “new” variant of superconducting amplifiers, considering that the first device obtained with  modern nanofab methods was demonstrated in 2012: Nat. Phys. 8, 623–627 (2012).  

**Response:** Removed.

**Summary**

 “As the amplifier is formed using the superconducting thin”. Please check language/grammar.  

**Response: **Corrected.

**Introduction**

 The list of references for experimentally demonstrated JTWPAs is not updated. Please check and possibly update the reference list according to state-of-the-art.

**Methods**

 “If a weak signal at frequency  is detected”. Confusing wording here: in order to get amplification, a weak signal should be 'applied' at input before being 'detected' at output.  

**Response: **It depends on application. For used as first stage detector such as in astronomy, the signal is indeed detected. But nevertheless, corrected to ‘applied’.

 Page 4 (end of first column): please check the correct use of punctuation, here and for the entire manuscript.  

**Response: **Done.

 Page 5: it would be interesting if the authors could provide more details on the computation time typically required for the full modelling of the single unit cell.  

**Response: **It varies between the many different topologies investigated here. It also depends on the meshing methods etc, but generally could be between tens of minutes to several hours.

 Page 5: in order to obtain the results reported in the figures of section 5, are equation (3) and (4) solved numerically? Or, an analytic solution, under certain conditions, is adopted? This is a very interesting aspect; however, it is not very clear in my opinion in the manuscript and should be clarified.  

**Response: **It's written in the text that these CMEs are solved numerically. Analytical solutions can be sought using the undepleted pump assumption as state in the text, but the procedures are rather standard but too lengthy to be included here (which can be found in the citation included), and is not important to the investigation we wish to perform here, which needs to include the effect of losses in the pump power.

 Fig 1: one may deduce that a second loading section is also included in the design, however this does not appear in Fig 1 sketch (only first and third loading sections are mentioned), can the authors please clarify?  

**Response: **We follow the convention used by Eom et. al. 2012, the very first paper that describe the scheme. The second loading section is in fact identical to the first, hence generally in the field, the loading are denoted as first loading and third loading to differentiate the two.

 Page 6: how is the contribution from the  estimated?  

**Response: **This was explained in the second box of Fig. 1, where we remove the superconductivity boundary condition of the model and calculate the wave vector which is then subtracted from the total wave vector of the model that include superconductivity.

 Methods: could the modelling method explained in section 2 be adapted for JTWPAs? it would be interesting if the authors could comment about this possibility.  

**Response: **Yes, indeed! We are currently preparing a paper to describe this effort.

 Please check that when an acronym is used for the first time, the definition is also reported. Example (MIKIDs in page 7).  

**Response: **The acronym is already explained at the end of introduction.

 Page 7: “From the plot, it clearly indicates that”. Please check language/grammar.  

**Response: **We believed this is fine.

**Section 3**

 How are plots (continuous lines) in fig from 2 to 8 obtained? If analytical expressions are used, can the authors please explicitly report the adopted analytical expressions for , ? Reporting the analytical expressions could help in possibly removing some of the figures (especially the ones showing secondary aspects, that can be equivalently well described with a formula).  

**Response: **It's calculated numerically using the Mattis-Bardeen equation which was explained in Section 2 and cited the source.

 Fig 6: The authors should add or at least discuss error bars for the experimental points.  

**Response: **We don’t have the information of the error bars for Fig.6a, as it is a one-off measurement that takes a large effort to perform experimentally.

 Page 10 “Therefore, it would be indicative to perform the same analysis at a fixed operational frequency instead of fractional frequency”. I totally agree with this. The authors could indeed focus directly on this kind of analysis (at fixed operational frequency) which is the useful one for the designing a device in a given frequency range. This will probably help in making the manuscript shorter and it will be easier for the reader to appreciate the key results.  

**Response: **Thanks. Equally, many peers would argue that it is easier to understand the analysis using fractional frequency. Therefore, we showed both for better comparison.

 Not necessary to repeat the acronym definition, the authors can define it just the first time that the acronym is used (general comment for all the manuscript). Example (KITWPA).  

**Response: **If the reviewer is referring to the repeated acronym definitions in the caption of figures and table, this is unfortunately the requirement from the journal, which is beyond the control of the authors.

 Page 16: “Therefore, we are confident that our proposed method can accurately predicts the gain-bandwidth performance of a KITWPA.” Very nice benchmark of the method. Does the same kind of validation check work for micro-strip geometries?  

**Response: **Thanks. Indeed, for microstrip as well. We report the validation in a separate article.

**Section 5.3**

 Is the adopted modelling method considering the frequency-dependence of dielectric losses? Can the authors please give more details of this aspect? This is particularly relevant for the micro-strip geometry.  

**Response: **Yes, it does. This is clearly shown Fig.17(b), and explained in Section 5.4 which focuses on the microstrip geometry.

**Section 5.3 **“if the signal-to-noise ratio of the incoming signal can be estimated a priori.” What does this exactly mean? How do the authors suggest doing that?

**Response:**  This section has been removed.

 Page 22 (last paragraph in first column):  This sentence is confusing, please check.

**Response:**  Done.

 Fig. 19: Very interesting comparison!  

**Response: **Thanks.

 Page 25: it would be useful if the authors could discuss this noise comparison also in terms of expected number of photons above the standard quantum limit for the TWPA added noise.  

**Response:** This paper focuses on the design consideration of KITWPA from the perspective of electromagnetism and thin film properties. The noise performance is a related but a different and important topic that needs equally in-depth investigations, which is beyond the scope of this paper.

 Page 25: The authors should be careful when comparing theoretical prediction with experimental results. For example, instead of using the expression “successfully achieve” I believe it would be more appropriate to say "are predicted to achieve"/"are expected to achieve" or similar expressions. I have the same language comment for the subsequent comparison with ref 22 and 7, since in the current manuscript no experimental data are presented.  

**Response: **Corrected.

# Reviewer report: Reviewer response for version 1

**DOI:** 10.21956/openreseurope.16055.r30186

**Published:** 2022-11-15

**Authors:** Wenlei Shan

**Affiliations:** 1 Advanced Technology Center (ATC), National Astronomical Observatory of Japan (NAOJ), Tokyo, Japan

This manuscript describes simulation, material properties, transmission line configuration, and design consideration of kinetic inductance traveling wave parametric amplifiers (TPAs). It is a comprehensive paper covering important aspects of development of TPAs, which is now gaining intensive interests both in quantum computing and week signal detection in astronomical observation. The applications will be more broadened with further revealing their features and potential. In this study, the authors established an improved simulation method by involving commercial EM simulator and, therefore, they can deal with circuit with various structures more realistically than before. They obtained new understandings in the selection of superconducting materials and transmission line configuration. With this set of new tools and consideration, they finished two designs of TPAs, which are expected to show superior performance. Apparently, this paper is valuable to the researchers who are doing study in this field.

 This review report will focus on some points that should be  improved in the way of presenting or should be further explained or analyzed. The manuscript can be better understood and more helpful to the society if these points are strengthened. The followings are major ones.

1.  Section 2, Methods. Equation (3) seems to be problematic. The equations should reduce to trivial ones dA/dz=0 if the nonlinearity approaches to zero. Normally, there should be the nonlinear factor on the left side of each equation, which is proportional to ( *I _p_/I*_0_) ^2^, where *Ip*  and *I*_0_ are pump current and critical current of the conducting strip respectively.  But the nonlinear factor is not found in Equation (3).
2. Section 2, Methods. The sequence of TPA simulation is explained in Figure 1. It is not difficult to understand how the ABCD matrix of each unit cell is obtained by using EM simulation. But it is not clear how these linear matrixes are combined with the coupled equations to calculate the gain-bandwidth profile of the TPA. It is obvious that the explanation of solving the nonlinear equations is missing. Readers will be unable to reproduce this calculation without knowing the method of solving the equations.
3. Section 2, All *R _s_* in the text and the figures are in unit of *k*Ω */sq*. They are unreasonably large sheet resistance and, therefore, the unit must be mistaken. 
4. Section 2, the ratio of surface inductance to resistance, *L _s_/R _s_* ,  is used as a figure of merit in selection of material for TPAs. This is reasonable if microstrip (MS) is used as the transmission line because the differential inductance of the line is dominated by *L _s_*. But for CPW, the geometric inductance is significant, therefore the propagation constant is less influenced by *L _s_* as in MS. In this case, the ratio of total inductance to Rs should be a better choice.
5. Section 2, Figure 5 presents the most important result that lead to one of the key conclusions of this paper, i.e. film normal state resistivity is not very critical in selection of material for TPAs. The authors consider these results are “rather unexpected”. At least, the observation of *L _s _* ∝ *ρ _N_/V _gap_* can be explained in the following way.  In the case of thin film, where the thickness is much less than the penetration depth, the film sheet impedance is approximately: 
. For the complex conductivity  of a superconductor, usually the real part is much smaller than the imaginary part. Then the surface resistance and reactance are approximated as ,  and , where t is the film thickness. The imaginary part of the conductivity can be expressed by using the energy gap for the (BCS) superconductor as , where  is normal state conductivity of the thin film, and Δ is the energy gap. Then the surface inductance *L _s _* can be expressed by energy gap and normal state resistivity  as . It is independent of frequency, proportional to film's normal state resistivity and reversely proportional to the energy gap. This is what is observed in Figure 5 b. However, from this analysis, *R _s_* is also proportional to  instead of  as observed in Figure 5 a. So, there is a contradiction between the theory presented here and the simulation result in Figure 5 (a) in the paper. Since the authors found the rule  from calculation, there must be a corresponding theory supporting it. It is necessary to provide the theoretical explanation of this key result and prove my analysis is wrong, which show a linear dependence. 
6. The ohmic loss of superconducting thin films are strongly dependent on the operating temperature. There are general missing of mentioning temperature information, at which the calculations were performed. This is a serious problem of the present version of this paper.
7. The authors claim that if the film thickness can be controlled accurately the selection of material is not critical. Because the film quality (for example the critical temperature) is dependent on the thickness especially when the film is very thin, only accurate thickness is not sufficient, and other properties much be maintained. 
8. The loss of transmission line is applied to the signal in the simulation. This makes the study more realistic than those where transmission loss is neglected. However, it is not clearly mentioned in the text whether the loss is also applied to the pump. Because the nonlinearity of the line is strongly dependent on the pumping strength, the dissipation of pump significantly affects the performance when the transmission line is physically long. Please clarify this point or add proper analysis on the impact of gradually weakening of the pump along the line. 

Followings are minor comments:

 Page 3, left column, line 11.  “…using the superconducting thin, we investigate...” missing word “film”.

 Page 3, right column, line 3. “In order to increase the interaction time.”  Please explain the definition of "interaction time"  or provide a reference. 

 Page 4, left column, line 12. “… amplified to preserve the total momentum of the system.”  Please explain “momentum of the system” or provide a reference. 

 Page 4, above equation (3), “Substituing the propagating wave into Equation 2, assuming that …”, please explain why these assumptions are reasonable especially the second one or provide references. 

 Page 5, left column, line 10 from bottom. “However, this approach neglects many subtle effects that could arise from cascading hundreds of these unit cells…” what are those subtle effects? or provide a reference. 

 Page 6, left column, the final line. “… which assume the kinetic inductance of the thin film is dominating in the case of TPA and hence the geometric inductance of the STL is neglected in the calculation…” What is neglected in most of calculations is the  magnetic field penetration into the film rather than that in dielectric media. Because films are normally thin, much thinner than the geometric scale of dielectric layer, the neglection of this part will not introduce significant effect. It is only a very small part of “geometric inductance”. 

 Page 13, left column, line 9 from bottom. “… using silicon monoxide (SiO) with a resistivity … loss tangent 0.004…”. The loss of SiO used here is obvious much larger than the more conventional used material  SiO _2_. Please explain the reason of making use of lossy material here. 

 Page 15,  left column, line 15. “Hence, even with a very high substrate resistivity of , the losses are still visibly higher due to the long transmission length.” What is the resistivity at cryogenic temperature? Most of thermally activated charges will get frozen at operation temperature. 

 Page 16, Figure 10. It is recommended to show a complete unit and specify (by labeling) the first and third loading sections and primary section corresponding to Figure 1. 

 Page 17, Figure 11 (b). It is recommended that the relative phase error Δϕ/ϕ should be used rather than the absolute value because the overall lengths are different in this comparison. 

 Page 21, left column, line 17. “Although generally one would assume that the amplifier should operate below the compression point, but this is not necessarily the case as long as there is still parametric gain; but the amplifier is not operating in the linear regime anymore.” This sentence does not make sense to me. 

 Page 21, Table 5. Please add the information of the total length of the line, for which the losses are calculated. 

 Page 24, Figure 18. The bridges cannot be clearly seen. An enlarged view is necessary.

 Page 25, left column, line 4. “we can estimate the noise power via  I2rmsR.” Is it better to use the thermal noise temperature , where L is the length, α is the real part of propagation constant, *k _B_* is Boltzmann constant and T is the physical temperature? It is not clear to me why the pump power is relevant. 

 Page 25, right column, line 2 from bottom. “…but in order to minimize cross talk…” cross talk issue is not discussed in this paper. It is suggested to add  analysis on cross talk, which will be interesting.

Is the study design appropriate and does the work have academic merit?

Yes

Is the work clearly and accurately presented and does it cite the current literature?

Partly

If applicable, is the statistical analysis and its interpretation appropriate?

Not applicable

Are all the source data underlying the results available to ensure full reproducibility?

Partly

Are the conclusions drawn adequately supported by the results?

Partly

Are sufficient details of methods and analysis provided to allow replication by others?

Partly

Reviewer Expertise:

Superconducting electronics, Radio Astronomical Techniques

I confirm that I have read this submission and believe that I have an appropriate level of expertise to confirm that it is of an acceptable scientific standard, however I have significant reservations, as outlined above.

# Response

**Published:** 2023-09-01

**Authors:** Boon Kok Tan

**Affiliations:** University of Oxford, UK

Dear Prof. Shan,   Thank you for taking your time to review the article thoroughly. My sincere apology for the long delay in responding. The responses for all your very useful comments are as below:  

 This manuscript describes simulation, material properties, transmission line configuration, and design consideration of kinetic inductance traveling wave parametric amplifiers (TPAs). It is a comprehensive paper covering important aspects of development of TPAs, which is now gaining intensive interests both in quantum computing and week signal detection in astronomical observation. The applications will be more broadened with further revealing their features and potential. In this study, the authors established an improved simulation method by involving commercial EM simulator and, therefore, they can deal with circuit with various structures more realistically than before. They obtained new understandings in the selection of superconducting materials and transmission line configuration. With this set of new tools and consideration, they finished two designs of TPAs, which are expected to show superior performance. Apparently, this paper is valuable to the researchers who are doing study in this field.

 This review report will focus on some points that should be improved in the way of presenting or should be further explained or analyzed. The manuscript can be better understood and more helpful to the society if these points are strengthened. The followings are major ones.

1.  Section 2, Methods. Equation (3) seems to be problematic. The equations should reduce to trivial ones dA/dz=0 if the nonlinearity approaches to zero. Normally, there should be the nonlinear factor on the left side of each equation, which is proportional to ( *I _p _/ I*_0_) ^2^ , where *I _p_*  and *I*_0_ are pump current and critical current of the conducting strip respectively.  But the nonlinear factor is not found in Equation (3).
**Response: **This is a very standard sets of coupled mode equation (CME) used widely for predicting the gain performance of KITWPA, except that we include the loss factor (\alpha) in the propagation constant (\gamma). See for example Eom et. al. 2012 for the similar derivation. The parameter Ip is directly related to the Ap in the equation.  
2. Section 2, Methods. The sequence of TPA simulation is explained in Figure 1. It is not difficult to understand how the ABCD matrix of each unit cell is obtained by using EM simulation. But it is not clear how these linear matrixes are combined with the coupled equations to calculate the gain-bandwidth profile of the TPA. It is obvious that the explanation of solving the nonlinear equations is missing. Readers will be unable to reproduce this calculation without knowing the method of solving the equations.
**Response:**As mentioned in the article, the unit cells are cascaded using transfer matrix method, which is a commonly used method. Basically, it’s just a dot-matrix product of the unit cells. This can be easily done using commercial or standard circuit emulators, or by writing a simple python code to do the matrix product. As pointed out in the text and in Fig.1, one can easily extract the propagation constant (\gamma) using the cascaded ABCD matrix, which are available in many electromagnetisms’ textbook e.g., Pozar’s Microwave Engineering. The CME noted in the article can then be solved numerically using python, Mathematica, Matlab or any other numerical software one prefers. As the article is focusing on the main message of the finding, these trivial step-by-step instructions would distract the focus of the reader on the scientific content of paper, hence not included. For these instructions, I invite you to look out for Joseph’s thesis which should be published shortly after he completed his PhD.  
3. Section 2, All R _s_ in the text and the figures are in unit of *k*Ω**** */ sq* . They are unreasonably large sheet resistance and, therefore, the unit must be mistaken. 
4. **Response:**Well spotted! Thanks for pointing that out. It was supposed to be mOhm not kOhm. All figures and texts are updated.  
5. Section 2, the ratio of surface inductance to resistance, *L _s_ / R _s_* ,  is used as a figure of merit in selection of material for TPAs. This is reasonable if microstrip (MS) is used as the transmission line because the differential inductance of the line is dominated by *L _s_* . But for CPW, the geometric inductance is significant, therefore the propagation constant is less influenced by *L _s_* as in MS. In this case, the ratio of total inductance to Rs should be a better choice. 
**Response: **As stated in the paper, the geometric inductance does not contribute to the wave-mixing process that promote amplification gain, hence it is more proper to use Ls instead of total inductance.
6. Section 2, Figure 5 presents the most important result that lead to one of the key conclusions of this paper, i.e. film normal state resistivity is not very critical in selection of material for TPAs. The authors consider these results are “rather unexpected”. At least, the observation of *L _s_* ∝ *ρ*_N_ */ V*_gap_ can be explained in the following way.  In the case of thin film, where the thickness is much less than the penetration depth, the film sheet impedance is approximately: . For the complex conductivity  of a superconductor, usually the real part is much smaller than the imaginary part. Then the surface resistance and reactance are approximated as*^^* , and , where t is the film thickness. The imaginary part of the conductivity can be expressed by using the energy gap for the (BCS) superconductor as , where  is normal state conductivity of the thin film, and Δ is the energy gap. Then the surface inductance *L _s_* can be expressed by energy gap and normal state resistivity  as . It is independent of frequency, proportional to film's normal state resistivity and reversely proportional to the energy gap. This is what is observed in Figure 5 b. However, from this analysis, *R _s_* is also proportional to  instead of  as observed in Figure 5 a. So, there is a contradiction between the theory presented here and the simulation result in Figure 5 (a) in the paper. Since the authors found the rule ** from calculation, there must be a corresponding theory supporting it. It is necessary to provide the theoretical explanation of this key result and prove my analysis is wrong, which show a linear dependence. 
**Response: **Thanks for pointing out the derivation. As you mentioned Rs=Xs(rho1/rho2). But as you pointed as well, there is an inverse relation between Xs and rho2, where rho2 = 1/(t Xs). If you replace this back to the Rs equation, it leads to Rs = rho1 t Xs^2 = rho1 t omega^2 Ls^2. Given that Ls is directly proportional to rhoN, you can see that in fact Rs is proportional to rhoN^2.  
7. The ohmic loss of superconducting thin films are strongly dependent on the operating temperature. There are general missing of mentioning temperature information, at which the calculations were performed. This is a serious problem of the present version of this paper.
**Response: **The operating temperature used for all the analysis presented in the paper is unchanged, and set at 10mK. This is highlighted at the end of the first paragraph of Section 3. We agree that the ohmic loss is dependent on the operating temperature, but to include the effect of various operating temperature is beyond the scope of this paper. A similar investigation on the effect of operating temperature can indeed be performed in the future.
8. The authors claim that if the film thickness can be controlled accurately the selection of material is not critical. Because the film quality (for example the critical temperature) is dependent on the thickness especially when the film is very thin, only accurate thickness is not sufficient, and other properties much be maintained. 
**Response:** We agree with the statement. However, in this paper, we focus only on the aspect of electromagnetism and superconductivity that affecting the performance of TWPA. The topic of fabrication complication addressing the other properties such as uniformity of the film etc., is beyond the scope of this paper.
9. The loss of transmission line is applied to the signal in the simulation. This makes the study more realistic than those where transmission loss is neglected. However, it is not clearly mentioned in the text whether the loss is also applied to the pump. Because the nonlinearity of the line is strongly dependent on the pumping strength, the dissipation of pump significantly affects the performance when the transmission line is physically long. Please clarify this point or add proper analysis on the impact of gradually weakening of the pump along the line. 
**Response:** As shown in Eq.(3), the term \alpha_p which denote the loss on the pump term is not omitted throughout, hence the loss is also applied to the pump. Furthermore, in paragraph 5 of Section 2, it is clear that the general CME presented in Eq.(3) do take into account the loss of the pump, as we pointed out that this CME can be reduced using the undepleted pump assumption as special case. This is also quite clearly shown in Fig.11(c). Nevertheless, we add in a line at the end of paragraph 5 to clearly pointing out to the readers that the effect of loss on the pump is included in all the analyses.

Followings are minor comments:  

**Response: **Thank you for your careful read of the article. Greatly appreciated.

 Page 3, left column, line 11.  “…using the superconducting thin, we investigate...” missing word “film”.  

**Response: **Done.

 Page 3, right column, line 3. “In order to increase the interaction time.”  Please explain the definition of "interaction time"  or provide a reference. 

**Response:**  As stated in the text, it is the interaction time between the pump and the signal tone within the nonlinear medium.

 Page 4, left column, line 12. “… amplified to preserve the total momentum of the system.”  Please explain “momentum of the system” or provide a reference.   

**Response: **There is no special definition of momentum used in the article, but the most basic notion of momentum used in all major physics textbook.

 Page 4, above equation (3), “Substituing the propagating wave into Equation 2, assuming that …”, please explain why these assumptions are reasonable especially the second one or provide references.   

**Response: **Reference provided.

 Page 5, left column, line 10 from bottom. “However, this approach neglects many subtle effects that could arise from cascading hundreds of these unit cells…” what are those subtle effects? or provide a reference.   

**Response: **Examples added.

 Page 6, left column, the final line. “… which assume the kinetic inductance of the thin film is dominating in the case of TPA and hence the geometric inductance of the STL is neglected in the calculation…” What is neglected in most of calculations is the  magnetic field penetration into the film rather than that in dielectric media. Because films are normally thin, much thinner than the geometric scale of dielectric layer, the neglection of this part will not introduce significant effect. It is only a very small part of “geometric inductance”.   

**Response: **We agree that the geometric inductance effect is minor in most cases, but it is more mathematically appropriate to include this effect in the calculation because the geometric inductance do not contribute to parametric gain, and may results in over-estimate the gain.

 Page 13, left column, line 9 from bottom. “… using silicon monoxide (SiO) with a resistivity … loss tangent 0.004…”. The loss of SiO used here is obvious much larger than the more conventional used material  SiO _2_. Please explain the reason of making use of lossy material here. 

**Response:**  SiO is chosen here just as an example, the analysis of this section remains the same if one replace SiO with other dielectric material. The effect of dielectric material is subsequently investigated in Section 5.4.

 Page 15,  left column, line 15. “Hence, even with a very high substrate resistivity of , the losses are still visibly higher due to the long transmission length.” What is the resistivity at cryogenic temperature? Most of thermally activated charges will get frozen at operation temperature.   

**Response: **Given that we do not possess the exact value of resistivity of various substrates at cryogenic temperature, it is difficult to perform the analysis with guess values for different substrates and hence resulting in misleading information. Nevertheless, we do agree that most thermally activated charges will get frozen to some degree at low operating temperature.

 Page 16, Figure 10. It is recommended to show a complete unit and specify (by labeling) the first and third loading sections and primary section corresponding to Figure 1.   

**Response: **Done.

 Page 17, Figure 11 (b). It is recommended that the relative phase error Δϕ/ϕ should be used rather than the absolute value because the overall lengths are different in this comparison. 

**Response:**  One important key for maximising the gain is that the total phase mismatch delta_phi is closed to zero, and this behaviour changes with the length of the device, exactly the point we would like to address in this plot. Therefore, it is adamant to show the absolute value on where it crosses the zero-point.

 Page 21, left column, line 17. “Although generally one would assume that the amplifier should operate below the compression point, but this is not necessarily the case as long as there is still parametric gain; but the amplifier is not operating in the linear regime anymore.” This sentence does not make sense to me. 

**Response:**  This section has been removed.

 Page 21, Table 5. Please add the information of the total length of the line, for which the losses are calculated.   

**Response: **Added.

 Page 24, Figure 18. The bridges cannot be clearly seen. An enlarged view is necessary.

**Response:**  Done.

 Page 25, left column, line 4. “we can estimate the noise power via a I2rmsR.” Is it better to use the thermal noise temperature , where L is the length,  is the real part of propagation constant, *k _B_* is Boltzmann constant and T is the physical temperature? It is not clear to me why the pump power is relevant. 

**Response:**  The majority of the current going through the TWPA is the pump, hence the relevance of pump power. This paragraph was written just to demonstrate relative different in power between the CPW and microstrip designs, the exact method used to estimate the noise power is not important for this purpose. The suggested method can be used as well, but the conclusion remains the same.

 Page 25, right column, line 2 from bottom. “…but in order to minimize cross talk…” cross talk issue is not discussed in this paper. It is suggested to add  analysis on cross talk, which will be interesting.  

**Response: **This is unfortunately beyond the scope of the paper, but I understand that it is important for other applications such as SIS mixers, which again is not the focus of this paper.

# Reviewer report: Reviewer response for version 1

**DOI:** 10.21956/openreseurope.16055.r29724

**Published:** 2022-08-15

**Authors:** Fausto Patricio Mena

**Affiliations:** 1 Electrical Engineering Department, University of Chile, Santiago, Chile; 2 National Radio Astronomy Observatory, Charlottesville, VA, USA

The work describes interesting methods and simulations for the implementation of Kinetic Inductance Travelling Wave amplifiers. There are three clear parts in the paper.

1. The first part presents an improvement in the Coupling Equations to model parametric amplifiers. Although the improvement seems to be intuitively correct, no clear justification is stated. Similar work towards modeling the effect of losses in parametric amplifiers have not been cited ( https://www.nrao.edu/meetings/isstt/papers/2019/2019063066.pdf).
2. The second part presents a meticulous study for finding and optimizing the most appropriate superconducting material suitable for implementing parametric amplifiers. This section is well written and very interesting insights are presented.
3. The last part presents several designs and simulations for future implementations of parametric amplifiers. Although the results are encouraging, my impression is that the authors are over-interpreting their findings. They have to be more careful when stating and drawing conclusions from simulations that have not been tested experimentally. Moreover, there is a stark contrast with the other sections, it needs a careful grammatical and spelling review.

The work, although interesting, it is rather lengthy and my recommendation is that the authors have to make more effort in shortening it, especially in Section 5. Sometimes the descriptions are repetitive. After presenting the first configuration, the other configurations do not need to be over-lengthy described since much of the findings are rather similar.

 More specific comments are given in the accompanying document. ( Boon-Kok Tan comments.pdf)

Is the study design appropriate and does the work have academic merit?

Yes

Is the work clearly and accurately presented and does it cite the current literature?

Partly

If applicable, is the statistical analysis and its interpretation appropriate?

Not applicable

Are all the source data underlying the results available to ensure full reproducibility?

Yes

Are the conclusions drawn adequately supported by the results?

Partly

Are sufficient details of methods and analysis provided to allow replication by others?

Yes

Reviewer Expertise:

NA

I confirm that I have read this submission and believe that I have an appropriate level of expertise to confirm that it is of an acceptable scientific standard, however I have significant reservations, as outlined above.

# Response

**Published:** 2023-09-01

**Authors:** Boon Kok Tan

**Affiliations:** University of Oxford, UK

Dear Prof. Mena,   Thank you for taking your time to review the article thoroughly. My sincere apology for the long delay in responding. The responses for all your very useful comments are as below:   The work describes interesting methods and simulations for the implementation of Kinetic Inductance Travelling Wave amplifiers. There are three clear parts in the paper.

1. The first part presents an improvement in the Coupling Equations to model parametric amplifiers. Although the improvement seems to be intuitively correct, no clear justification is stated. Similar work towards modeling the effect of losses in parametric amplifiers have not been cited ( https://www.nrao.edu/meetings/isstt/papers/2019/2019063066.pdf).
**Response:** Thanks for the suggestion. Citation added.
2. The second part presents a meticulous study for finding and optimizing the most appropriate superconducting material suitable for implementing parametric amplifiers. This section is well written and very interesting insights are presented.
**Response:** Thanks.
3. The last part presents several designs and simulations for future implementations of parametric amplifiers. Although the results are encouraging, my impression is that the authors are over-interpreting their findings. They have to be more careful when stating and drawing conclusions from simulations that have not been tested experimentally. Moreover, there is a stark contrast with the other sections, it needs a careful grammatical and spelling review.  
**Response: **This paper focuses on the design considerations of KITWPA from the perspective of electromagnetism mainly, hence all the conclusions were made referring to the simulations done with rigorous mathematical frameworks, not via experimental results which certainly would have to include even more subtle effects such as fabrication defects etc, which would be beyond the scope of this paper. We toned done the ‘over-interpretation’, nevertheless, in the updated version as suggested.  

The work, although interesting, it is rather lengthy and my recommendation is that the authors have to make more effort in shortening it, especially in Section 5. Sometimes the descriptions are repetitive. After presenting the first configuration, the other configurations do not need to be over-lengthy described since much of the findings are rather similar.  

**Response: **The updated manuscript has been shortened.

 More specific comments are given in the accompanying document. ( Boon-Kok Tan comments.pdf)   The responses were given in the pdf.
